# Highly plastic genome of *Microcystis aeruginosa *PCC 7806, a ubiquitous toxic freshwater cyanobacterium

**DOI:** 10.1186/1471-2164-9-274

**Published:** 2008-06-05

**Authors:** Lionel Frangeul, Philippe Quillardet, Anne-Marie Castets, Jean-François Humbert, Hans CP Matthijs, Diego Cortez, Andrew Tolonen, Cheng-Cai Zhang, Simonetta Gribaldo, Jan-Christoph Kehr, Yvonne Zilliges, Nadine Ziemert, Sven Becker, Emmanuel Talla, Amel Latifi, Alain Billault, Anthony Lepelletier, Elke Dittmann, Christiane Bouchier, Nicole Tandeau de Marsac

**Affiliations:** 1 Institut Pasteur, Pasteur Genopole ®, F-75015, Paris, France; 2 Institut Pasteur, Unité des Cyanobactéries; CNRS, URA2172, F-75015, Paris, France; 3 INRA, UMR CARRTEL, BP 511, 74203 Thonon cedex, France; 4 Aquatic Microbiology, Institute for Biodiversity and Ecosystem Dynamics, University of Amsterdam, Nieuwe Achtergracht 127, 1018 WS Amsterdam, The Netherlands; 5 Institut Pasteur, Unité de Biologie Moléculaire du Gène chez les Extrêmophiles; F-75015, Paris, France; 6 Université d'Aix-Marseille, Laboratoire de Chimie Bactérienne, CNRS-UPR9043, 31 chemin Joseph Aiguier, 13402 Marseille cedex 20, France; 7 Humboldt-Universität zu Berlin, Institut für Biologie, Molekulare Ökologie, Chausseestr. 117, 10115 Berlin, Germany; 8 Netherlands Institute of Ecology NIOO-KNAW, Centre for Limnology, Rijksstraatweg 6, 3631 AC Nieuwersluis, The Netherlands; 9 Genoscope – Centre National de Séquençage, 2 rue Gaston Crémieux CP5706 91057 Evry cedex, France; 10 Department of Genetics, Harvard Medical School, Boston MA 02115, USA

## Abstract

**Background:**

The colonial cyanobacterium *Microcystis *proliferates in a wide range of freshwater ecosystems and is exposed to changing environmental factors during its life cycle. *Microcystis *blooms are often toxic, potentially fatal to animals and humans, and may cause environmental problems. There has been little investigation of the genomics of these cyanobacteria.

**Results:**

Deciphering the 5,172,804 bp sequence of *Microcystis aeruginosa *PCC 7806 has revealed the high plasticity of its genome: 11.7% DNA repeats containing more than 1,000 bases, 6.8% putative transposases and 21 putative restriction enzymes. Compared to the genomes of other cyanobacterial lineages, strain PCC 7806 contains a large number of atypical genes that may have been acquired by lateral transfers. Metabolic pathways, such as fermentation and a methionine salvage pathway, have been identified, as have genes for programmed cell death that may be related to the rapid disappearance of *Microcystis *blooms in nature. Analysis of the PCC 7806 genome also reveals striking novel biosynthetic features that might help to elucidate the ecological impact of secondary metabolites and lead to the discovery of novel metabolites for new biotechnological applications. *M. aeruginosa *and other large cyanobacterial genomes exhibit a rapid loss of synteny in contrast to other microbial genomes.

**Conclusion:**

*Microcystis aeruginosa *PCC 7806 appears to have adopted an evolutionary strategy relying on unusual genome plasticity to adapt to eutrophic freshwater ecosystems, a property shared by another strain of *M. aeruginosa *(NIES-843). Comparisons of the genomes of PCC 7806 and other cyanobacterial strains indicate that a similar strategy may have also been used by the marine strain *Crocosphaera watsonii *WH8501 to adapt to other ecological niches, such as oligotrophic open oceans.

## Background

Dated approximately 3 billion years old by fossil records, cyanobacteria were the first oxyphototrophic prokaryotes present on Earth [1]. As architects of the Earth's atmosphere they had a major impact on the evolution of aerobic metabolism and the evolution of life [2]. Cyanobacteria still play a fundamental role in the functioning of global ecosystems by significantly contributing to carbon fluxes [3,4] and by providing nitrogen used for primary production [5]. On the other hand, cyanobacterial blooms may lead to a loss of biodiversity in the phytoplanktonic communities and, by generating very high quantities of organic matter used by anoxygenic bacteria in the bottom layers of water resources, can cause massive death of fish by asphyxia [6]. The financial costs resulting from cyanobacterial proliferations are considerable (*e.g. *200 million Australian dollars/year in Australia) [7].

Freshwater cyanobacteria of the genus *Microcystis *are distributed worldwide, and are involved in numerous proliferation events in stratified lakes [8]. In their natural environment, *Microcystis *cells are organized in large colonies of various sizes and shapes, which were used to define various morphospecies. Five of these have recently been reunified as a single species, *Microcystis aeruginosa *[9]. The determinism of the morphogical variations within this polymorphic cyanobacterial species is currently under debate.

The ecology of *M. aeruginosa *is characterized by an annual life cycle comprising a spring and summer pelagic phase, and an overwintering benthic phase [10]. During the pelagic phase, *M. aeruginosa *colonies migrate daily in the water column [11] and may accumulate to form blooms or scums on the surface of the water. Thus, on a daily basis, as well as during the benthic and pelagic phases, colonies are exposed to changing environmental conditions of light, temperature and oxygen concentrations.

In the last decade, cyanobacterial blooms have been involved in numerous cases of animal [12] and human [13] poisonings, mainly due to the ability of *Microcystis *cells to synthesize toxins, in particular variants of microcystin [14]. Many other oligopeptides, such as cyanopeptolins, aeruginosins, microginins, microviridins and cyclamides may also be produced [15]. Other peptides and congeners doubtless remain to be discovered, as do their respective biosynthesis pathways.

To gain further insight into the ecophysiology of *Microcystis aeruginosa*, we deciphered the genome sequence of the toxic strain PCC 7806. The results presented here associate descriptive genomics and comparisons with the genomes of other cyanobacteria isolated from freshwater and marine ecosystems to highlight the ecophysiological peculiarities of this strain, and put its particularly high genome plasticity into a cyanobacterial context.

## Results and discussion

### General features of the *M. aeruginosa *PCC 7806 genome

The 12× shotgun sequencing project produced 90,000 sequence reads, and their assembly resulted in more than 500 contigs. After the first steps of a long finishing process performed using CAAT-Box [16] and Consed [17] software, the number of contigs was reduced to 328 (N50 = 100kb), 116 of which were more than 3,000 bases in length (up to 533,374 bases). The genome contains an unusually high number of long DNA repeats. Most of the extremities of these contigs consist of DNA repeated sequences including gene coding for transposases (see below). The 116 contigs were deposited in the EMBL database (AM778843–AM778958). The genome sequence of *M. aeruginosa *PCC 7806 (Mic-PCC7806), represented by these contigs, consists of 5,172,804 bases, with an average G+C content of 42%. These values are consistent with those previously determined using thermally denatured DNA [18]. The contigs were annotated using CAAT-Box software and a total of 5,292 predicted protein-coding sequences (CDSs) were validated manually. These CDSs were compared to several protein (Uniprot, COG and 45 cyanobacterial proteomes) and motif databases (Prosite and Pfam).

All the genomes used for the comparative studies described below are listed in the Methods section.

### Comparison with other cyanobacterial genomes

**Figure 1:**
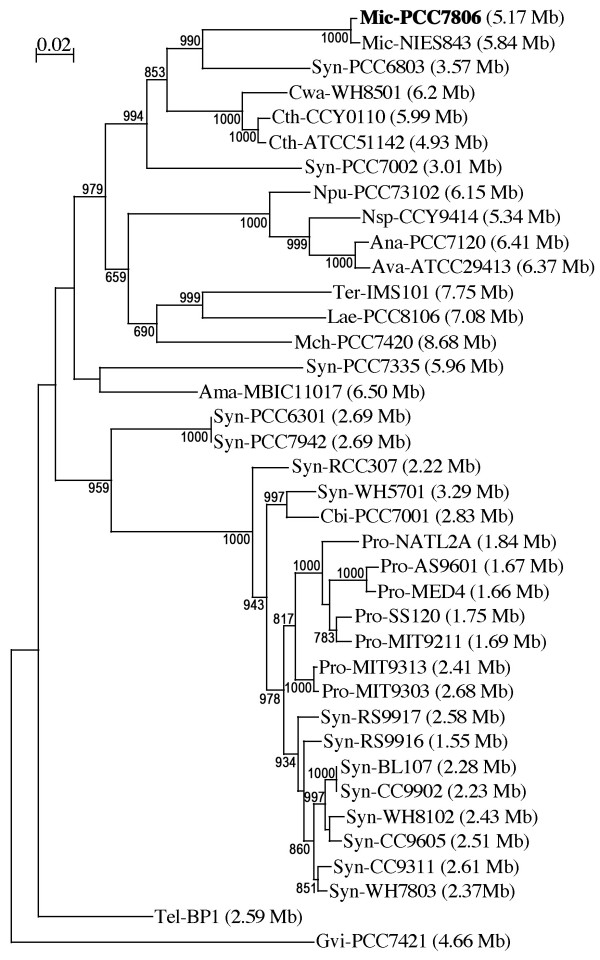
**Phylogenetic maximum likelihood (ML) tree based on the concatenated 23S-16S rDNA sequences of diverse cyanobacterial lineages.** The sequences were taken from public databases. Strain identifiers, and the methods used for the phylogenetic analysis, are described in the Methods section. The scale bar represents the average number of nucleotide substitutions per site. Genome sizes in megabases (Mb) are mentioned in parentheses. Trees were constructed using three methods (ML, Neighbor Joining and Maximum Parsimony). ML bootstrap values are indicated only if the bootstrap values obtained with the three methods are > 500 (1000 resamplings).

A concatenated dataset of large and small subunit rRNA sequences (23S and 16S rRNA) was used to construct a phylogenetic tree including Mic-PCC7806 and 37 other cyanobacterial strains (Figure 1). The tree is congruent with previously published ones based on 16S rRNA sequences [19,20], but shows higher statistical support at most nodes (especially internal ones), probably due to the larger number of positions used. The strains of the genus *Microcystis *form a well-supported group (BV of 853‰) with *Synechocystis *sp. (Syn-PCC6803), *Crocosphaera watsonii *(Cwa-WH8501) and *Cyanothece *sp. (Cth-CCY0110 and Cth-ATCC51142). Within this group, *Microcystis *is most closely related to Syn-PCC6803 (BV of 990‰).

The Mic-PCC7806 genome was compared to the recently publicly available genome of *Microcystis aeruginosa *strain NIES-843 (Mic-NIES843) [21]. Although the average similarity between the orthologous genes is 94%, their comparison emphasizes that the two genomes largely differ both in length and gene composition (Table 1). Indeed, the Mic-NIES843 genome is 0.6 Mb longer than that of Mic-PCC7806. Moreover, the two genomes display a high number of strain-specific genes (838 for Mic-PCC7806 and 1760 for Mic-NIES843). Interestingly, most of these genes are absent from 44 other cyanobacterial complete genomes suggesting that they have recently been acquired in each of the two *Microcystis *strains independently. Although the two genomes contain the same proportions of large DNA repeats (~12%, see below), their distribution and size partly differ since Mic-PCC7806 contains 48 repeats longer than 3,000 bases for only 11 in Mic-NIES843. The comparison of the location of similar genes in the largest contig of the Mic-PCC7806 assembly (contig328) and in the Mic-NIES843 genome shows numerous genomic rearrangements (see Additional file 1). These rearrangements, probably facilitated by the presence of large repeats, render the Mic-NIES843 genome of little help for the finishing of the assembly process of the Mic-PCC7806 genome sequence.

**Table 1:** Comparison between two *Microcystis *genomes

Strain (a)	Mic-PCC7806	Mic-NIES843
Genome length	5.17 Mb (116 Contigs)	5.84 Mb
rRNA loci	2	2
tRNA loci	41	42
Number of CDSs	5292	6312
Putative transposases (COG similarity)	362 (6.8%)	469 (7.4%)
Proteins linked by BDBH	3322 (63%)	3322 (53%)
Proteins absent in the other *Microcystis *genome (b)	838 (16%)	1760 (28%)
Strain-specific proteins (c)	644/838 (76%)	1484/1760 (84%)
Large repeats (d)	11.7%	11.7%

**Figure 2:**
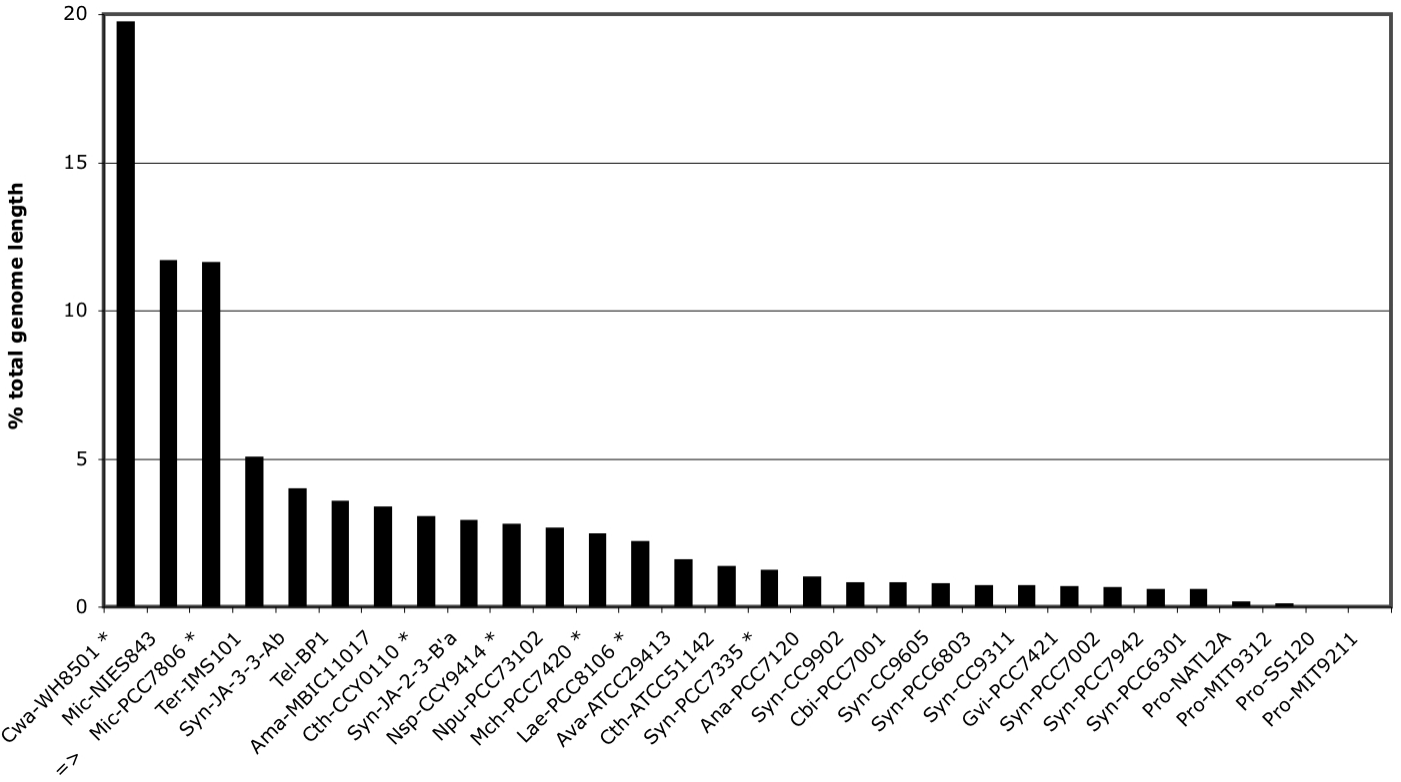
**Percentage of DNA repeated sequences in the total genome length.** This analysis was performed on complete and in-finishing (*) cyanobacterial genomes. The strain identifiers are listed in the Methods section. Only DNA repeats containing more than 1000 bases, and with an identity threshold >90%, are taken into account.

(a) See the Methods section for the strain identifiers.

(b) Proteins that do not share similarity (> 40%) with any proteins in the other *Microcystis *genome.

(c) Proteins with no similarity (> 40%) with any proteins in the 44 other cyanobacterial genomes.

(d) Proportion of large repeats (> 1000 bases; > 90% identity) in the genome (see Figure 2).

Mb: megabases; CDS: coding sequence; COG: cluster of orthologs; BDBH: bidirectional best hit.

The 5292 CDSs of the Mic-PCC7806 genome were also compared to the proteomes of 44 strains representing the diversity of the cyanobacterial lineages (all publicly available genomes excluding Mic-NIES843). The distribution of the best High Scoring Pairs (HSPs) found using Blastall software indicates a high similarity between the proteome of Mic-PCC7806 and a group of three strains Cth-ATCC51142, Cth-CCY0110 and Cwa-WH8501 (Table 2). This is puzzling, since Mic-PCC7806 is closer to Syn-PCC6803 than to this group in the 23S-16S phylogeny (Figure 1). In order to exclude possible bias introduced by uneven distribution of CDSs in these genomes, we analyzed only the orthologs shared by three of these genomes, Mic-PCC7806, Syn-PCC6803 and Cwa-WH8501. Based on BiDirectional Best Hit (BDBH) analyses, 1789 CDSs of the Mic-PCC7806 genome were found to correspond to putative orthologs in Cwa-WH8501 and Syn-PCC6803. The mean Blast score of these CDSs was 381 for the comparison between Mic-PCC7806 and Cwa-WH8501, and only 366 for Mic-PCC7806 versus Syn-PCC6803. The distribution curve of all the Blast scores (see Additional file 2) showed that the Mic-PCC7806 genome was more closely related to Cwa-WH8501 than to Syn-PCC6803 for all score values considered. The absence of congruence between the results obtained with rDNA sequences and the core proteins means that additional data sets for other members of these three cyanobacterial genera are required. Nevertheless, the results obtained by comparing all the orthologous genes shared by Mic-PCC7806 (freshwater strain) and Cwa-WH8501 (marine strain) are consistent with the fact that freshwater and marine cyanobacteria are interspersed in global 16S rDNA phylogenetic trees [20].

**Table 2:** Distribution of the best Blastp of the Mic-PCC7806 proteome against other cyanobacterial proteomes

No Significant HSP	14.4%
Cth-ATCC51142	15.5%
Cth-CCY0110	13.4%
Cwa-WH8501	9.9%
Mch-PCC7420	9%
Npu-PCC73102	5.8%
Syn-PCC6803	5.1%
Ana-PCC7120	4.3%
Nsp-CCY9414	4.2%
Lae-PCC8106	4%
Ava-ATCC29413	3.9%
Ama-MBIC11017	2.5%
Ter-IMS101	1.95%
Syn-PCC7002	1.6%
Gvi-PCC7421	1.15%
Syn-PCC7335	1%
Syn-PCC7942	0.5%
Tel-BP1	0.4%
Syn-WH5701	0.2%
Syn-JA-2-3B'a	0.1%
Syn-PCC6301	0.1%
Other genomes	0%

See the Methods section for the strain identifiers. HSP: High-Scoring Segment Pair.

Three distinct groups of proteins were identified on the basis of Blastp analyses of the 5,292 CDSs of Mic-PCC7806, with a selection of 15 other cyanobacterial genomes displaying at least 1% of best Blastp hits with Mic-PCC7806 (Table 2). The composition of these groups largely depends on the threshold chosen to consider that two proteins are similar. Without an obvious breakpoint in the distribution of protein similarities between different genomes (see Additional file 2), we arbitrarily chose a threshold of 40% of similarity, considering that below this value two proteins do not share the same function. The three groups are as follows:

- The "maeru40" group included 764 CDSs (14.4%) specific to the Mic-PCC7806 genome and not found in the 15 selected genomes; 438 (8.3%) of them have no homolog in the uniprot database;

- The "core40" group comprised 652 proteins (12.3%) sharing significant Blastp scores with at least one CDS in each of the 15 other genomes tested;

- The last group, designated "other40", consisted of 3,876 CDSs (73%) sharing significant Blastp scores with CDSs in only some of the other 15 genomes tested.

The small percentage of CDSs in the core40 group reflects the wide diversity of the cyanobacterial genomes analyzed. In the other40 group, the distribution of the Mic-PCC7806 CDSs among the tested genomes matches their phylogenetic distances based on 23S-16S rDNA sequences. For example, in this group, 10% of the CDSs were present in all the genomes, apart from that of Gvi-PCC7421, which is the most distant phylogenetically (Figure 1). Moreover, the four closest genomes to Mic-PCC7806 (Syn-PCC6803 and the group including Cwa-WH8501, Cth-CCY0110 and Cth-ATCC51142) appear to have the same percentage (2%) of CDSs, shared only with Mic-PCC7806.

### Plasticity of the genome of *M. aeruginosa *PCC 7806

#### Large number of long repeated sequences

The Mic-PCC7806 genome includes a very large number of DNA sequences containing more than 1000 bases that are repeated at least twice in the genome with more than 90% identity. A comparative analysis of all the cyanobacterial genome sequences available in databases showed that Mic-PCC7806, Mic-NIES843 and Cwa-WH8501 are particularly rich in such DNA repeats. Indeed, they account for 11.7%, 11.7% and 19.8% of the total DNA length, respectively (Figure 2). The cumulative size of the DNA repeated sequences is not strictly a function of genome length as Mic-PCC7806 and Cwa-WH8501 genomes have the highest percentage of DNA repeats, but are of intermediate size relative to the other cyanobacterial genomes (see Additional file 3). In the Mic-PCC7806 genome, 1346 CDSs (25%) are located within these DNA repeats. Among these CDSs, only 256 and 92 belong to the maeru40 and core40 groups, respectively. Most of the CDSs of the core40 group correspond to orthologs that are not located within DNA repeats in other cyanobacterial genomes. This implies that over the course of evolution, resident genes were probably captured by genetic mobile elements. A large number of CDSs (362) are very similar to transposases from the COG database, and 93% of them are located within long DNA repeated sequences. At least 46 transposases correspond to ISMae*1A/2/3/4 *that had previously been characterized in strain PCC7806 [22], but a large majority of the other transposases cannot be clearly associated with any known insertion sequence (only 17 are associated to IS*30*, 7 to IS*1 *and 3 to IS*5*). The genome of Cwa-WH8501 also contains numerous putative transposases. One third of them are associated to IS*5*, but none to IS*30*; the DNA repeated sequences are therefore different in each genome, and cannot account for the close phylogenetic relationship between these two strains.

#### Synteny of cyanobacterial genomes

**Figure 3:**
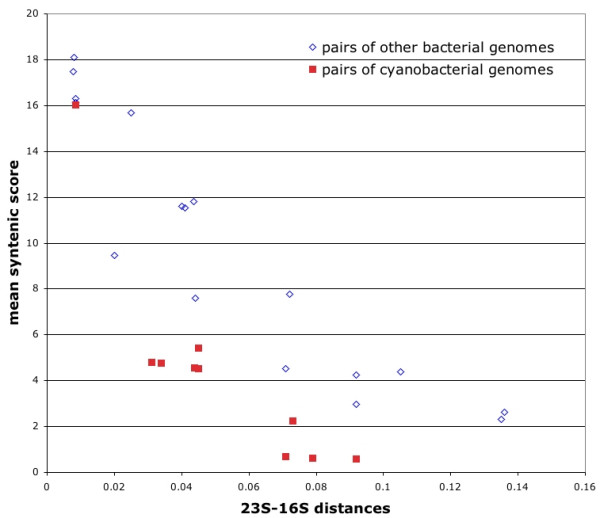
**Comparison of the syntenic scores of cyanobacterial genomes (filled square) and other bacterial genomes (empty diamond) according to the maximum likelihood distances of their 23S-16S sequences calculated by Phyml (see Methods).** The pairs of cyanobacterial genomes used in this study are listed in the Methods section.

Although Mic-PCC7806 and Mic-NIES843 are very closely related strains (Figure 1), their genomes contain a high number of rearrangements. Moreover, an unexpectedly low level of synteny was also observed between the *Microcystis *strains and two close relatives, Cwa-WH8501 and Syn-PCC6803 (68% mean CDS similarity). Since the same observation was made for all the cyanobacterial genomes tested, we compared the dynamics of these genomes using a large set of other bacterial genomes chosen on the basis of their sizes and phylogenetic distances. To this end, a synteny score was calculated for a number of genome pairs (see Methods), and then compared to their evolutionary distance based on the 23S-16S rDNA tree. This analysis showed that the synteny scores for cyanobacterial genomes were significantly lower than those obtained for pairs of non-cyanobacterial genomes with similar genome lengths and 23S-16S phylogenetic distances (Figure 3). Similar results were obtained for all the cyanobacterial genomes tested. This means that the low synteny scores observed cannot be related to the long DNA repeated sequences, which occur only in the Mic-PCC7806 and Cwa-WH8501 genomes. These results are in agreement with those of Fang *et al. *[23], who showed that both persistent and rare genes are significantly clustered in most of the 169 bacterial genomes analyzed. However, in a minority subset of bacterial genomes that includes the cyanobacteria, persistent genes were found to be fairly uniformly distributed throughout the genome.

Interestingly, only 8 clusters with at least 4 CDSs remain syntenic in the genomes of Mic-PCC7806, Cwa-WH8501 and Syn-PCC6803. Four of these clusters correspond to ribosomal proteins. The other clusters are shown in Table 3. Considering the very low level of synteny between cyanobacterial genomes, it is likely that these specific clusters have been subjected to strong positive selection pressure and may play essential roles in these cyanobacteria. Some of these clusters are clearly linked to a specific biological function, such as the transport of phosphate (see Additional file 4) [24], while others consist of conserved proteins with unknown functions. One can thus speculate that these proteins may be involved in the same biological pathway as their close neighbors.

**Table 3:** Conserved gene clusters in the genomes of Mic-PCC7806, Cwa-WH8501 and Syn-PCC6803


**Cluster 1 = Phosphate transport system**								
Gene name	*sphX*	*pstS*	*pstC*	*pstA*	*pstB1*	*pstB2*		
Mic-PCC7806 contig328	*mic3546*	*mic3547*	*mic3548*	*mic3549*	*mic3550*	*mic3552*		
Cwa-WH8501 contig3	*EAM51827.1*	*EAM51828.1*	*EAM51829.1*	*EAM51831.1*	*EAM51832.1*	*EAM51833.1*		
Syn-PCC6803	*sll0679*	*sll0680*	*sll0681*	*sll0682*	*sll0683*	*sll0684*		
Gene annotation	periplasmic phosphate-binding protein of ABC transporter	phosphate-binding periplasmic protein precursor	phosphate transport system permease protein	phosphate transport system permease protein	phosphate transport ATP-binding protein	phosphate transport ATP-binding protein		
								
**Cluster 2 = Ci-concentrating mechanism**								
								
Gene name	*ccmK2*	*ccmk1*	*ccmL*	*ccmM*	*ccmN*			
Mic-PCC7806 contig303	*mic5495*	*mic5496*	*mic6196*	*mic5695*	*mic5233*			
Cwa-WH8501 contig2	*EAM52133.1*	*EAM52134.1*	*EAM52135.1*	*EAM52136.1*	*EAM52137.1*			
Syn-PCC6803	*sll1028*	*sll1029*	*sll1030*	*sll1031*	*sll1032*			
Gene annotation	carbon dioxide concentrating mechanism protein	carbon dioxide concentrating mechanism protein	putative carboxysome assembly protein	putative carboxysome structural protein	putative carboxysome assembly protein			
								
**Cluster 3 = Unassigned function**								
								
Gene name		*yidC*		*rnpA*				
Mic-PCC7806 contig303	*mic5398*	*mic5399*	*mic5400*	*mic6364*				
Cwa-WH8501 contig2	*EAM51643.1*	*EAM51642.1*	*EAM51641.1*	*EAM51640.1*				
Syn-PCC6803	*slr1472*	*slr1471*	*slr1470*	*slr1469*				
Gene annotation	COG1847 Predicted RNA-binding protein	COG0706 Preprotein translocase subunit	No similarity; highly conserved in cyanobacteria	protein subunit of ribonuclease P				
								
**Cluster 4 = ATP synthase**								
								
Gene name/Alternate gene name	*atpC*	*atpA*	*atpH/atpD*	*atpF*	*atpG*	*atpE/atpH*	*atpB/atpI*	*atpI*
Mic-PCC7806 contig290	*mic4443*	*mic4444*	*mic4445*	*mic4446*	*mic4447*	*mic4448*	*mic4449*	*mic4451*
Cwa-WH8501 contig1	*EAM53207.1*	*EAM53206.1*	*EAM53205.1*	*EAM53204.1*	*EAM53203.1*	*EAM53202.1*	*EAM53201.1*	*EAM53200.1*
Syn-PCC6803	*sll1327*	*sll1326*	*sll1325*	*sll1324*	*sll1323*	*ssl2615*	*sll1322*	*sll1321*
Gene annotation	ATP synthase gamma chain	ATP synthase alpha chain	ATP synthase delta chain of CF(1)	ATP synthase B chain (subunit I) of CF(0)	ATP synthase of B' chain (subunit b') of CF(0)	ATP synthase C chain of CF(0)	ATP synthase A chain	ATP synthase protein I

For each of the three genomes, the gene identifiers are indicated in italics. See the Methods section for the strain identifiers.

#### Intergenic regions

**Figure 4:**
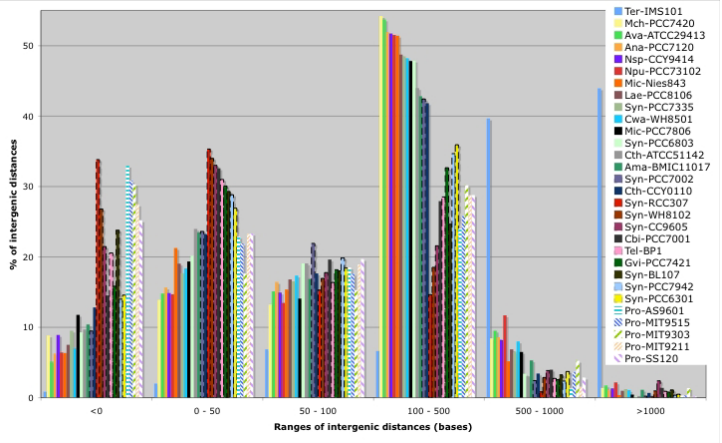
**Distribution of the intergenic distances in diverse cyanobacterial genomes.** The distances are based on the public syntaxic annotation of each genome. Strain identifiers are listed in the Methods section.

Four groups can clearly be identified among the cyanobacterial genomes studied on the basis of their intergenic distances (Figure 4). The first consists solely of the genome of Ter-IMS101, which harbors exceptionally long intergenic regions. To the best of our knowledge, no data has been published on this genome, which makes it impossible to rule out the possibility that these regions result from the poor quality of the sequence or the syntaxic annotation. The second group includes the genome of Mic-PCC7806 and, among others, those of Cwa-WH8501 and Syn-PCC6803 which have a high proportion of intergenic sequences around 300 bases long; in the case of the Mic-PCC7806 genome, less than 35% of intergenic sequences are shorter than 100 bases. The third group comprises the genomes of Syn-PCC7942, Tel-BP1 and Gvi-PCC7421, which have short intergenic regions, similar in size to those found in a number of other bacterial genomes (see Additional file 5). The fourth group includes some members of the *Prochlorococcus *genus that have very small genomes with short or no intergenic regions.

The mean length of the intergenic sequences seems to be linked to the genome size of the cyanobacterium, except for the genome of Syn-PCC6803, which is smaller (3.6 Mb) than that of Gvi-PCC7421 (4.6 Mb), but harbors longer intergenic sequences. Although the role of long intergenic sequences in most cyanobacterial genomes remains unclear, we can surmise that they might be involved in the modulation of gene expression, which would allow cells to acclimate to rapid environmental changes.

#### Cluster of atypical genes

In order to explore the plasticity of the Mic-PCC7806 genome further, the number of CDSs with an atypical dinucleotide composition was determined using a one-order Markov chain-based methodology [25]. This method can identify genes that may have been acquired recently by lateral transfers. In the Mic-PCC7806 genome, a total of 1971 atypical genes were found, including 1402 within 159 clusters of atypical genes (CAGs) that probably correspond to recently acquired foreign genomic elements (Table 4). As expected, more than 98% of Mic-PCC7806 genes belonging to the core40 group were not in CAGs, and 31% of the atypical genes were in the maeru40 group. Moreover, a high percentage (80%) of the transposase genes were in CAGs (16% of the genes present in CAGs encode putative transposases). Compared to seven other cyanobacterial genomes, those of Mic-PCC7806 and Mic-NIES843 harbor the highest percentages of atypical genes (37%) and CAGs (34% and 36%, respectively). These findings may indicate that the *Microcystis *genomes contain a higher proportion of genes recently acquired by lateral transfers than the other genomes studied.

**Table 4:** Analysis of the presence of atypical genes in several cyanobacterial genomes

**Strain (a)**	**Number of genes**	**Number of AGs**	**Number of CAGs**	**Number of genes in CAGs**	**AGs in CAGs**
Mic-PCC7806	5292	1971 **(37%)**	**159**	1790 (34%)	**78%**
Mic-NIES843	6364	**2335 (37%)**	126	**2298 (36%)**	66%
Cwa-WH8501	5967	1004 (17%)	61	523 (9%)	60%
Mch-PCC7420	**7357**	2008 (27%)	150	1403 (19%)	68%
Syn-PCC6803	3314	494 (15%)	32	243 (7%)	75%
Npu-PCC72103	6182	1259 (20%)	65	596 (10%)	64%
Lae-PCC8106	6142	1549 (25%)	102	1084 (18%)	67%
Ana-PCC7120	5430	1254 (23%)	48	390 (7%)	69%

(a) See the Methods section for the strain identifiers. Higher scores are shown in bold. AG: Atypical gene; CAG: Cluster of atypical genes.

#### Putative restriction and modification systems

Blast searches for restriction enzymes and examination of genes surrounding DNA methylases, identified 21 potential restriction enzymes (see Additional file 6), seven of which were found to be co-localized with putative methylases (see Additional file 7) in the Mic-PCC7806 genome. The Mic-NIES843 genome also contains a high number (at least 17) of putative restriction enzymes [21]. Blast searches revealed that 14 restriction enzymes are common to both genomes. In contrast, seven and eight restriction enzymes seem specific to Mic-PCC7806 and Mic-NIES843, respectively. The *Microcystis aeruginosa *strains might thus constitute a rich source of novel restriction enzymes potentially useful in biotechnology. According to Zhao *et al. *[26], filamentous cyanobacteria (*Anabaena*, *Spirulina *and *Nostoc *strains) contain more restriction and modification genes than unicellular cyanobacteria (*Synechocystis*, *Synechococcus *and *Prochlorococcus *strains). Based on COG annotations, at least as many restriction-modification genes were found in Mic-PCC7806, Mic-NIES843 and Cwa-WH8501 as in filamentous cyanobacteria. Thus, rather than corresponding to a difference between filamentous and unicellular cyanobacteria, the restriction-modification gene content of *Microcystis aeruginosa *may reflect the potential exposure of the cells to high concentrations of foreign DNA due to the presence of numerous other bacterial cells or viruses associated with *Microcystis *colonies [27]. This exposure to foreign DNA is also consistent with the high number of CAGs putatively acquired by lateral transfers. Whether such a hypothesis might also hold true for planktonic cyanobacteria of the genus *Crocosphaera *remains an open question.

In bacterial genomes containing a high number of genes for restriction enzymes, short palindromic sequences corresponding to the target sites of these enzymes may be under-represented [28]. Since the genomes of *Microcystis aeruginosa *and Cwa-WH8501 contain a very high number of putative restriction enzymes, there should be a number of under-represented short sequences that correspond to restriction sites. To test this hypothesis, the number of occurrences of each 6-mer was counted, and a frequency distribution calculated for Mic-PCC7806, Mic-NIES843, Cwa-WH8501 and Syn-PCC6803 (Table 5). The under-represented sites in the three first genomes were not found in Syn-PCC6803, a genome devoid of restriction enzymes [29], supporting the idea that these rare 6-mers could indeed correspond to restriction enzyme sites. In total, there are 4096 possible 6-mers, 1.5% of which are palindromes. Fifty-one percent of the rarest 1% of 6-mers in the Mic-PCC7806 genome are palindromes (see Additional file 8). Palindromes are thus over-represented among the rarest 6-mers, further supporting the hypothesis that they could correspond to sites cut by restriction enzymes. The identity of the rarest 1% of 6-mers in the Mic-PCC7806 genome was compared to known restriction sites in other organisms as identified by New England Biolabs [30]. We found that 20 of the 41 sites corresponded to sites cut by restriction enzymes in other organisms.

**Table 5:** Distribution of rare 6-mers in cyanobacterial genomes

Ratio (a) Obs/Shuf	Number of 6-mers			
	
	Mic-PCC7806	Mic-NIES843	Cwa-WH8501	Syn-PCC6803
< 0.02	**6 (4)**	**12 (8)**	**6 (3)**	**0 (0)**
< 0.04	13 (8)	11 (5)	9 (3)	7 (3)
< 0.06	10 (4)	8 (3)	6 (3)	5 (2)
< 0.08	2 (1)	2 (1)	3 (2)	6 (1)
< 0.1	2 (1)	3 (0)	13 (6)	14 (1)

(a) Ratio between the frequency observed (Obs) for a given 6-mers and the frequency for the same 6-mers after shuffling (Shuf) of the genome sequence.

Figures in bold represent the number of 6-mers present 50× more in the shuffled sequence than in the original sequence. Figures in parentheses indicate the number of palindromic sites.

See the Methods section for the strain identifiers.

A novel DNA modification system was discovered recently in the Gram-positive bacterium *Streptomyces lividans *66 [31]. This system results in the degradation of DNA *in vitro *by oxidative, double-stranded, site-specific cleavage during electrophoresis, and is determined by a cluster of five genes (*dndA-B-C-D-E*). The *dnd *gene products incorporate sulfur into the DNA backbone as a sequence-selective, stereospecific phosphorothioate modification [32]. According to He *et al. *[33], the resistance of phosphorothiate linkages to a variety of nuclease activities, and the site specific nature of such a modification suggest that phosphorothioates could have a role comparable to that of DNA methylation in protection against nucleases. Although the presence of *dndB *homologs is not clear in the genomes of cyanobacteria, the rest of the cluster was found in several of them including Mic-PCC7806 (see Additional file 9). Despite the low level of synteny in cyanobacterial genomes (see above), the *dndC-D-E *genes are still clustered.

### Unraveling genetic features related to the ecophysiology of *M. aeruginosa *PCC 7806

#### Life cycle, colony formation and floatation

During the overwintering benthic phase of their life cycle, *Microcystis *colonies withstand long periods of darkness. A fermentation pathway has been proposed based on biochemical data [34]. All the genes coding for the enzymes required for the various steps in this pathway have been identified in the genome sequence (see Additional file 10). During the benthic phase, *Microcystis *colonies are exposed to lower temperature and higher pressure. In this respect, it is interesting to note the presence of a gene (*mic5251*) coding for a protein similar to Hik33 that perceives osmotic stress and cold stress in Syn-PCC6803 [35]. Another gene, *mic5237*, is similar to the Ana-PCC7120 *orrA *gene whose product is involved in osmoregulation [36]. A genomic island carrying *actM *and *pfnM*, two genes that encode eukaryotic-like proteins, actin and profilin (an actin cognate binding partner), respectively, have been discovered in the Mic-PCC7806 genome. As shown by Guljamow *et al*. [37], this eukaryotic-like actin forms a shell-like structure that could strengthen cell resistance to hydrostatic and osmotic pressures. Interestingly, these genes are only present in *Microcystis *cells that inhabit the Braakman water reservoir (The Netherlands), which was cut off from the sea in the 20^th ^century, and from which the Mic-PCC7806 strain was originally isolated.

Although several different *M. aeruginosa *morphotypes have been described [38], little is known about their colony formation. The genome sequence of strain Mic-PCC7806 revealed a gene coding for a lectin (*mvn*; *mic3128*), which binds specifically to a sugar moiety present on the surface of Mic-PCC7806 cells, and a binding partner has been identified in the lipoplysaccharide fraction [39]. A functional correlation between the potent toxin microcystin and this lectin has been demonstrated, with possible implications for the formation of colonial aggregates that are characteristic of different *Microcystis *morphotypes. Another protein, MrpC (microcystin-related protein C), has been shown to be a potential target of an O-glycosyltransferase of the SPINDLY family [40]. *In situ*, this protein accumulates at the cell surface, and is involved in cellular interactions. Microcystins may therefore have an impact on the aggregation of *Microcystis *cells, which is very important for the competitive advantage of these organisms over other phytoplankton species. Mvn and MrpC are predominantly encoded in toxic strains [[38] and E. Dittmann, unpublished data], but not in the genome of Mic-NIES843. The latter strain may thus represent an ecotype that differs from Mic-PCC7806 in the characteristics of the cell surface. Genes coding for a Ser/Thr kinase (*mic0129*) and a Ser/Thr phosphatase of the PPP family (*mic4622*) are found within two clusters that may be involved in cell wall synthesis. Mic-PCC7806 also has two genes that encode Wzc-like protein Tyr kinases (*mic2086 *and *mic1089*) and three genes coding for Wzb-like protein Tyr phosphatases (*mic3515*, *mic3588 *and *mic6566*). In *E. coli*, the function of these systems is known to be related to the synthesis of the cell wall and polysaccharides [41]. These kinases/phosphatases could potentially be involved in colony formation. Colony migration depends not only on the cell ballast resulting from the accumulation of photosynthates and the size of the colonies, but also on the synthesis of gas vesicles (GV), intracellular structures providing cells with buoyancy [42]. The Mic-PCC7806 genome carries a cluster of 12 genes required for GV synthesis, two of which, *gvp*V and *gvp*W, are novel [43]. The *mic1271 *and *mic1270 *genes are highly similar to the genes coding for a light-regulated two-component system in Syn-PCC6803. This system, which consists of a cyanobacterial phytochrome (Cph1) and its response regulator (Rcp1), has been proposed to play a role in the control of processes required for the adaptation from light to dark conditions and *vice-versa *[44]. Moreover, all the genes involved in circadian rhythm [45] are present in Mic-PCC7806 (see Additional file 11). Whether day-night cycles and the timing of vertical migration of *Microcystis *colonies in the water column are controlled by this phytochrome and by the circadian clock mechanism would be worth being tested.

In natural populations of *Microcystis*, oxidative stress was shown to induce programmed cell death (PCD) [46]. Accordingly, 5 putative eukaryotic caspase-like genes were identified by PSI-Blast in the genome of strain Mic-PCC7806. Three of them (Mic0980, Mic3930 and Mic4051) showed best similarity with Mic-NIES843 proteins that lack caspase-like motifs. Consequently, these three proteins are likely involved in other functions than PCD. In contrast, the Mic1068 protein showed similarity in the caspase-like region with one protein of Mic-NIES843 (MAE24870). The last caspase-like protein of Mic-PCC7806 (Mic5406) is strain-specific. Both *mic1068 *and *mic5406 *are expressed, and a cross-reaction with human caspase-3 polyclonal antisera was observed indicating that the proteins are synthesized (data not shown). Alignment of the regions containing the conserved caspase domains of Mic1068, Mic5406, MAE24870 and a yeast metacaspase shows that the Histidine-Cysteine catalytic diad of the key functionnal regions of the capases is conserved (see Additional file 12). PCD might thus be triggered when *Microcystis *cells are exposed to severe environmental stress conditions, leading to the rapid decline of blooms, as has been suggested by Berman-Frank *et al. *in the case of Ter-IMS101 [47]. Mic-PCC7806 and Mic-NIES843 are the only unicellular cyanobacteria known to have genes coding for HstK-like kinases (*mic1879 *and *mic1015*), proteins characterized by the presence of both His and Ser/Thr kinase domains [48,49]. Some of these kinases are implicated in either the iron homeostasis/oxidative stress response or in the differentiation of N_2_-fixing cells in filamentous cyanobacteria [[48,49] and C-C Zhang, unpublished data]. Cell differentiation does not occur in *M. aeruginosa*, but it would be interesting to test whether these HstK-like protein kinases are involved in iron homeostasis and/or in the control of programmed cell death in response to oxidative stress. It has been proposed that the methionine recycling pathway may contribute to preventing oxidative stress in *Bacillus subtilis *[50,51]. Interestingly, all the genes involved in this pathway are present in the Mic-PCC7806 genome (see Additional file 13). One of these genes, *mtnW *(*rbcL*_IV_), encodes a 2,3-diketo-5-methylthiopentyl-1-phosphate enolase that has been identified in all the *Microcystis *strains tested including Mic-NIES843 [21,52], but not in other cyanobacteria for which the genome sequences are available, except Lae-PCC8106 (accession n° ZP_01618990) and Cth-PCC8801 (accession n° ZP_02940034). The putative methionine recycling pathway may thus have a specific role related to the lifestyle or ecological niches inhabited by members of the genera *Microcystis*, *Lyngbya *and *Cyanothece*.

#### Genetic potential for the production of secondary metabolites

Cyanobacteria are known as prolific producers of natural products, in particular of the nonribosomal peptide and polyketide classes [15,53]. However, the potential to produce complex secondary metabolites largely varies among the cyanobacterial genera and species, and even among individual strains. Remarkably, the genomes of Mic-PCC7806, Mic-NIES843 and Cwa-WH8501 differ from unicellular cyanobacteria of other genera in that they contain a large number of genes that encode nonribosomal peptide synthetases (NRPS) and polyketide synthases (PKS). Interestingly, such genes in Mic-PCC7806 outnumber those found in Mic-NIES843 and Cwa-WH8501 (Table 6). Apart from the terrestrial filamentous strain Npu-PCC73102, Mic-PCC7806 devotes the largest percentage of its genome (~3.5%) to secondary metabolite production (Table 6) [54].

**Table 6:** Gene clusters involved in the biosynthesis of secondary metabolites

Strain (a)	Size Mb	% SM (b)	PKS				NRPS		Patellamide like
			
			Modular type I	Iterative type I/glycolipid synthase (c)	Enedyine type	PKS III	NRPS/PKS	NRPS	
Mic-PCC7806	5.2*	3.5	1	0	1	1 (d)	2	1	1
Mic-NIES843	5.8	2.6	1	0	1	0	2	1	0
Cwa-WH8501	6.2*	1.6	0	0	0	0	1	6 (e)	0
Npu-PCC73102	8.2	4.5	2	2	0	0	6	1	0
Syn-PCC6803	3.6	0	0	0	0	0	0	0	0

(a) See the Methods section for the strain identifiers.

(b) %SM: percentage of the genome dedicated to secondary metabolites of the non-ribosomal peptide, polyketide or patellamide type family.

(c) Iterative PKS I not including enedyine type.

(d) PKS III is associated with modular PKS I.

(e) NRPS clusters in Cwa-WH8501 are all of a small size with an average of 1–2 genes per cluster.

* in-finishing genome; Mb: megabases; PKS: polyketide synthase; NRPS: non-ribosomal peptide synthetase.

**Figure 5:**
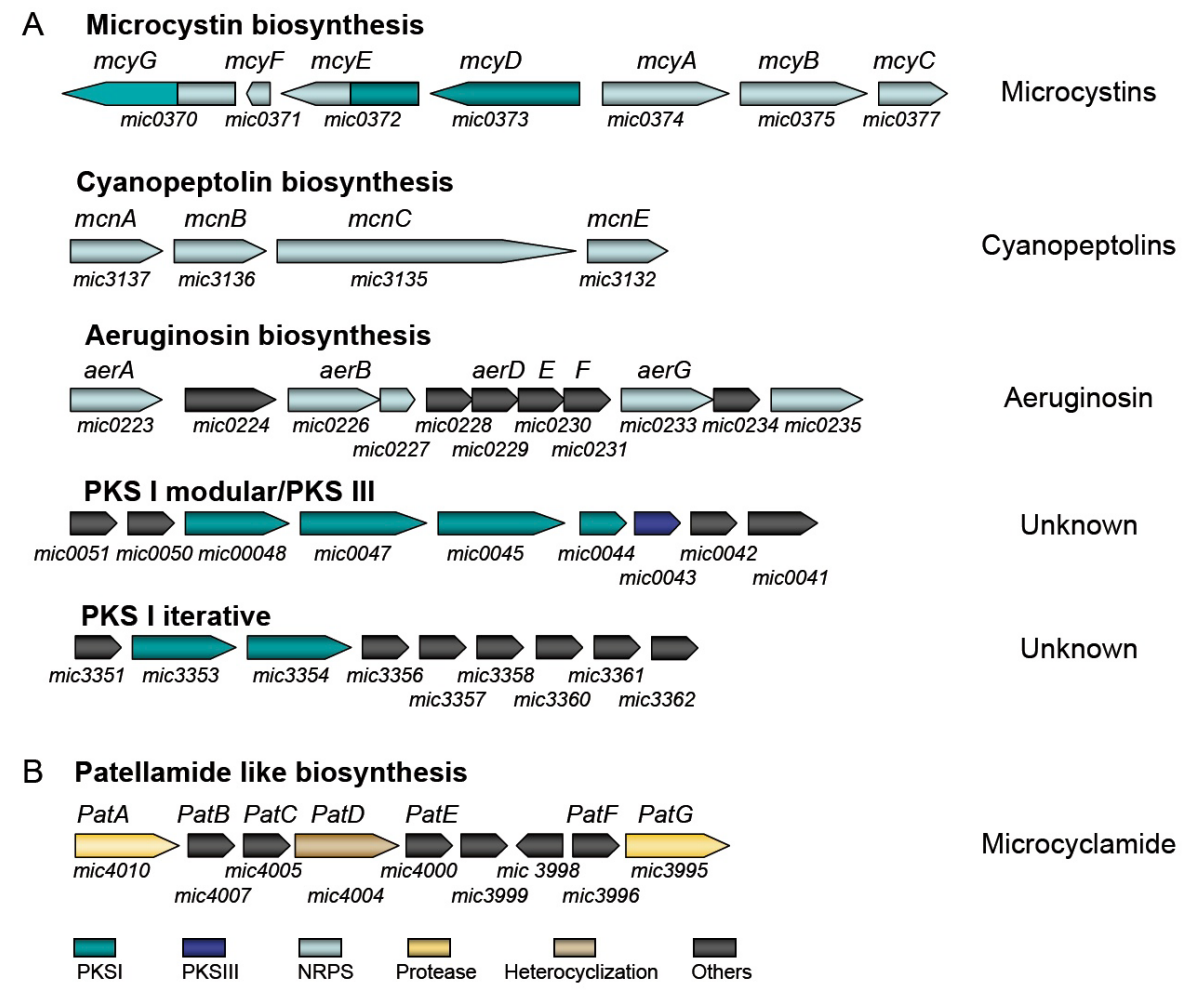
**Schematic representation of secondary metabolite gene clusters in Mic-PCC7806.** (A) Gene clusters encoding non-ribosomal peptide synthetases (NRPS) and polyketide synthases (PKS). The names assigned to individual genes in Mic-PCC7806, or to genes that were characterized in other cyanobacterial strains are indicated above the arrows. Products assigned to the respective pathways are shown on the right. (B) Gene cluster encoding enzymes potentially involved in a patellamide-like pathway. Names of patellamide biosynthesis genes are indicated above the arrows. Gene identifiers in the Mic-PCC7806 genome are indicated below the arrows.

The strain Mic-PCC7806 is known to produce two isoforms of microcystin [55]. The corresponding genes in the bi-directional *mcyA-J *gene cluster encoding NRPS, PKS and tailoring enzymes [56,57] could be re-assigned during the genome sequencing project (Figure 5). Genes for cyanopeptolin biosynthesis (*mcn *cluster) could be assigned based on the amino acid specificities of the substrate-activating domains of a second NRPS gene cluster that was congruent with the amino acid moieties contained in the cyanopeptolin structure [58] (Figure 5). The *mcn *genes of Mic-PCC7806 display some similarity to the anabaenopeptilide genes of *Anabaena *strain 90 [59] and to the cyanopeptolin genes of *Microcystis wesenbergii *[60]. In addition, the genome of Mic-PCC7806 harbors three NRPS and PKS gene clusters (Figure 5). One of the clusters displays some similarity to the cluster involved in the production of the protease inhibitor aeruginoside in *Planktothrix agardhii *Cya 126 [61]. The genomic data therefore clearly indicate that strain Mic-PCC7806 might be capable of producing a variant of aeruginosin (Figure 5).

The two remaining PKS I gene clusters do not show significant similarity to any known cyanobacterial biosynthetic gene clusters, and may be involved in the production of hitherto unknown compounds (Figure 5 and Table 6). The first gene cluster encodes an iterative PKS I that is similar in both architecture and sequence to the PksE of various actinobacteria, and is accompanied by several tailoring enzymes including three halogenases. The actinobacterial enzyme is involved in the biosynthesis of enedyine type antitumor antibiotics [62]. The second PKS gene cluster encodes a modular PKS I complex accompanied by several putative tailoring enzymes, and a PKS III type enzyme that is capable of synthesizing compounds of the chalcone/stilbene family. These biosynthetic enzymes are widespread in plants but have only recently been discovered in bacteria [63]. A comparison of the biosynthetic potential of Mic-PCC7806 and Mic-NIES843 reveals that three of the large NRPS/PKS complexes, namely those dedicated to microcystin, cyanopeptolin and aeruginosin production, are encoded on both genomes, whereas some other gene clusters are not shared by both genomes. The biosynthetic versatility of members of the genus *Microcystis *may thus be larger than expected, since the two strains selected for genome sequencing have similar chemotypes. Beside the NRPS and PKS encoding genes, the genome of Mic-PCC7806 contains a gene cluster similar to the patellamide genes that were recently detected in symbiotic cyanobacterial strains of ascidians [64]. Patellamides are a family of cyclic peptides generated from a ribosomally-synthesized precursor. Mic-PCC7806 is the first freshwater cyanobacterium showing the capability to produce patellamide-like peptides. A peptide with striking similarity to the patellamides, microcyclamide, has been reported in *M. aeruginosa *strain NIES-298 [65]. Chemical analyses have revealed that the gene cluster discovered in Mic-PCC7806 is indeed dedicated to the production of a microcyclamide-type compound [66]. The genome of Mic-PCC7806 could attract further attention, as it also contains gene clusters comprising unique features that have yet to be characterized and which may well produce so-far unidentified natural substances.

Transporter genes are commonly found in the immediate vicinity of the secondary metabolite biosynthetic genes. These secondary metabolites may therefore at least partly function at the surface of *Microcystis *cells, in the colony-surrounding sheath or in their planktonic environment. Gene clusters involved in the synthesis of secondary metabolites are frequently associated with genes that confer resistance to these metabolites, which would otherwise be toxic to the cells producing them. In Mic-PCC7806, only the transport system associated with the uncharacterized PKS I/PKS III hybrid compound (Figure 5) shows any similarity to typical efflux transporters that potentially confer self-resistance. The compound produced could therefore have an allelopathic or antibacterial role in the environment [67].

## Conclusion

Among bacteria, members of the genus *Microcystis *have a particularly high potential for the production of complex secondary metabolites, although this is lower than that of some actinobacterial and myxobacterial genomes that have been shown to devote up to 10% of their coding capacity to the production of secondary metabolites [68]. Genomics has already been useful to the study of secondary metabolites, and has restored natural product research as a major field of pharmaceutical research [69]. Analysis of the Mic-PCC7806 genome has revealed striking novel biosynthetic features that might help to explain the ecological impact of these compounds, as well as guide the search for novel metabolites of biotechnological importance.

Data mining of the genome sequence of Mic-PCC7806 has also shed light on genes that are of importance for the colonial life style and survival of this cyanobacterium in its natural habitat, either during the benthic phase or when it forms blooms on the surface of the water. One of the most intriguing features of this genome is its exceptional plasticity, characterized by a very large number of long repeated sequences, and genes encoding transposases and putative restriction enzymes. These biological entities may generate deletions, duplications, conversions, and rearrangements in the chromosome [70]. One illustration of these changes is the marked loss of synteny between this genome and other cyanobacterial genomes. In addition, the presence of a large number of clustered atypical genes in the genome of Mic-PCC7806 suggests that frequent gene acquisition events by lateral transfers have occurred.

Genome plasticity in prokaryotes is often considered to be an adaptive strategy allowing microorganisms to promote diversification in a way similar to sexual reproduction in eukaryotic organisms. However, genomic rearrangements can also impede the co-expression of genes [71] and disrupt gene dosage effects [70]. The resulting trade-off between gene conservation and rearrangement in the chromosome depends on various factors and processes linked to the ecophysiology of the microorganisms. The cost of chromosome rearrangements may be greater for fast-growing bacteria, than for slow-growing ones such as cyanobacteria [72]. The relative importance of the process of gene co-expression in cyanobacteria is more difficult to evaluate. However, it is worth noting that some of the eight syntenic clusters found in Mic-PCC7806 concern transport systems for nutrients, such as phosphate, which is often the limiting factor in marine and freshwater ecosystems.

Although Syn-PCC6803, Cwa-WH8501 and Mic-PCC7806 are phylogenetically closely related, only the last two strains have highly plastic genomes containing high proportions of long DNA repeats and transposase genes. No obvious explanation can be deduced from the ecophysiological features of these two strains. Indeed, members of the genus *Microcystis *are freshwater colonial cyanobacteria that proliferate in eutrophic ecosystems (e.g. ≤ 2.10^7^cells/ml in [73]) while the *Crocosphaera *are marine nitrogen-fixing cyanobacteria living in oligotrophic open oceans (≤ 10^3 ^cells/ml [74]). *Microcystis *colonies may display chaotic population dynamics, with alternating explosion and crash phases [75], but to the best of our knowledge, no such data are available for *Crocosphaera*. Such chaotic population dynamics could explain the widespread occurrence of rearrangements in the Mic-PCC7806 genome, if, as proposed by Helm *et al. *[76] for *Salmonella *serovars, bottlenecks and genetic drifts generally promote the fixation of mildly harmful rearrangements.

More genome sequences of members of the *Microcystis *and *Crocosphaera *genera are required to clarify the molecular basis of their genome plasticity, at both the intergeneric and intraspecies levels. This will also provide a deeper understanding of the evolutionary significance of this mode of adaptation to the environment. The ongoing sequencing of such genomes should make it possible to reach this goal in the near future. More generally, large cyanobacterial genomes constitute excellent model systems for studying genome dynamics and the mechanism(s) by which some gene clusters may escape rearrangement and retain the same physical organization in several different lineages.

## Methods

### Strain and genome nomenclature

#### Abbreviations used to designate the cyanobacterial strains (genome accession number)

Ama-MBIC11017: *Acaryochloris marina *MBIC11017 (embl: CP000828)

Ana-PCC7120: *Anabaena*/*Nostoc *sp. PCC 7120 (embl: BA000019)

Ava-ATCC29413: *Anabaena variabilis *ATCC 29413 (embl: CP000117)

Cbi-PCC7001: *Cyanobium *sp. PCC 7001 (gb: 1106012173546)

Cth-ATCC51142: *Cyanothece *sp. ATCC 51142 (embl: CP000806)

Cth-CCY0110: *Cyanothece *sp. CCY0110 (gb: 1101676644636–1101676644658)

Cwa-WH8501: *Crocosphaera watsonii *WH8501 (embl: AADV02000100)

Syn-JA33Ab: *Cyanobacteria Yellowstone *JA-3-3Ab (embl: CP000239)

Syn-JA23B'a: *Cyanobacteria Yellowstone *JA-2-3B'a (embl: CP000240)

Gvi-PCC7421: *Gloeobacter violaceus *PCC 7421 (embl: BA000045)

Lae-PCC8106: *Lyngbya aestuari *PCC 8106 (gb: 1099428180563–1099428180584)

Mch-PCC7420: *Microcoleus chthonoplastes *PCC 7420 (gb:1103659003780–1103659003836)

Mic-NIES843: *Microcystis aeruginosa *NIES-843 (embl: AP009552)

Mic-PCC7806: *Microcystis aeruginosa *PCC 7806 (embl: AM778843–AM778958)

Nsp-CCY9414: *Nodularia spumigena *CCY9414 (gb:1099428179735–1099428179797)

Npu-PCC73102: *Nostoc punctiforme *PCC 73102 (kindly provided by J. C. Meeks) [77]

Pro-SS120: *Prochlorococcus marinus *SS120 (embl: AE017126)

Pro-AS9601: *Prochlorococcus marinus *AS9601 (embl: CP000551)

Pro-MED4: *Prochlorococcus marinus *MED4 (embl: BX548174)

Pro-MIT9211: *Prochlorococcus marinus *MIT9211 (embl: AALP01000001)

Pro-MIT9215: *Prochlorococcus marinus *MIT9215 (embl: CP000825)

Pro-MIT9301: *Prochlorococcus marinus *MIT9301 (embl: CP000576)

Pro-MIT9303: *Prochlorococcus marinus *MIT9303 (embl: CP000554)

Pro-MIT9312: *Prochlorococcus marinus *MIT9312 (embl: CP000111)

Pro-MIT9313: *Prochlorococcus marinus *MIT9313 (embl: BX572095)

Pro-MIT9515: *Prochlorococcus marinus *MIT9515 (embl: CP000552)

Pro-NATL1A: *Prochlorococcus marinus *NATL1A (embl: CP000553)

Pro-NATL2A: *Prochlorococcus marinus *NATL2A (embl: CP000095)

Syn-BL107: *Synechococcus *sp. BL107 (gb: 1099739244347)

Syn-CC9311: *Synechococcus *sp. CC9311 (embl: CP000435)

Syn-CC9605: *Synechococcus *sp. CC9605 (embl: CP000110)

Syn-CC9902: *Synechococcus *sp. CC9902 (embl: CP000097)

Syn-PCC6301: *Synechococcus elongatus *PCC 6301 (embl: AP008231)

Syn-PCC7002: *Synechococcus *sp. PCC 7002 (embl: CP000951)

Syn-PCC7335: *Synechococcus *sp. PCC 7335 (gb: 1103496006889–1103496006899)

Syn-PCC7942: *Synechococcus elongatus *PCC 7942 (embl: CP000100)

Syn-RCC307: *Synechococcus *sp. RCC307 (embl: CT978603)

Syn-RS9916: *Synechococcus *sp. RS9916 (gb: 1100013018508)

Syn-RS9917: *Synechococcus *sp. RS9917 (gb: 1099465004208)

Syn-WH5701: *Synechococcus *sp. WH5701 (gb: 1099465003749–1099465003864)

Syn-WH7803: *Synechococcus *sp. WH7803 (embl: CT971583)

Syn-WH7805: *Synechococcus *sp. WH7805 (gb: 1099646010155–1099646010157)

Syn-WH8102: *Synechococcus *sp. WH8102 (gb: BX548020)

Syn-PCC6803: *Synechocystis *sp. PCC 6803 (embl: BA000022)

Tel-BP1: *Thermosynechococcus elongatus *BP-1 (embl: BA000039)

Ter-IMS101: *Trichodesmium erythreum *IMS101 (embl: CP000393)

#### Pairs of cyanobacterial genomes used in Figure 3

Mic-PCC7806/Cwa-WH8501

Mic-PCC7806/Syn-PCC6803

Cwa-WH8501/Syn-PCC6803

Lae-PCC8106/Ter-IMS101

Npu-PCC73102/Ava-ATCC29413

Npu-PCC73102/Ana-PCC7120

Npu-PCC73102/Nsp-CCY9414

Nsp-CCY9414/Ana-PCC7120

Ava-ATCC29413/Nsp-CCY9414

Ana-PCC7120/Ava-ATCC29413

#### Other bacterial strains used in Figure 3 (genome accession number)

*Shigella dysenteriae*, serovar 1, strain Sd97/Sd197 (CP000034_GR)

*Acidovorax avenae *subsp. *citrulli *AAC00-1 (NC_008752)

*Agrobacterium tumefaciens *str. C58 (NC_003062)

*Bacillus subtilis *subsp. *subtilis *str. 168 (NC_000964)

*Bordetella parapertussis *12822 (NC_002928)

*Escherichia coli *APEC O1 (NC_008563)

*Enterobacter *sp. 638 (NC_009436)

*Janthinobacterium *sp. Marseille (NC_009659)

*Klebsiella pneumoniae *subsp. *pneumoniae *MGH 78578 (CP000647)

*Listeria monocytogenes *EGD-e (NC_003210)

*Methylococcus capsulatus *str. Bath (NC_002977)

*Ochrobactrum anthropi *ATCC 49188 chromosome 1 (NC_009667)

*Polaromonas naphthalenivorans *CJ2 (NC_008781)

*Pseudomonas aeruginosa *PA7 (NC_009656)

*Pseudomonas fluorescens *PfO-1 (NC_007492)

*Rhizobium etli *CFN 42 (NC_007761)

*Rhizobium leguminosarum *bv. *viciae *3841 (NC_008380)

*Rhodobacter sphaeroides *ATCC 17025 (NC_009428)

*Rhodoferax ferrireducens *T118 (NC_007908)

*Shewanella loihica *PV-4 (NC_009092)

*Shewanella oneidensis *MR-1 (NC_004347)

*Shewanella *sp. W3-18-1 (NC_008750)

*Shigella boydii *Sb227 (NC_007613)

*Silicibacter *sp. TM1040 (NC_008044)

*Yersinia enterocolitica *subsp. *enterocolitica *8081 (NC_008800)

*Yersinia pestis *CO92 (NC_003143)

*Photorhabdus luminescens *subsp. *laumondii *TTO1 (NC_005126)

### DNA preparation and sequencing

The strain *Microcystis aeruginosa *PCC 7806 (kept in constant culture since its isolation in 1978; Pasteur Culture Collection, Paris, France [18]) was grown as described [52]. The genome sequence of Mic-PCC7806 was determined by a whole-genome shotgun strategy. Two libraries were generated using genomic DNA extracted with the kit Nucleobond AGX500 (Macherey-Nagel, Hoerdt, France) and shared by nebulization. The first library contained inserts from 1 to 4 kb cloned in pcDNA2.1 (Invitrogen Life Technologies, Carlsbad, CA, USA) and the second included inserts from 5 to 8 kb cloned in the low-copy vector pSYX34 (gift of F. Kunst, Institut Pasteur, Paris, France). A BAC library was constructed into the vector pBeloBAC11 (inserts ≤ 20 kb) (Epicentre, Madison, USA) using spooled DNA extracted as described [78] and partially hydrolyzed with *Hin*dIII.

Plasmid DNA purification was performed using the Montage Plasmid Miniprep96 Kit (Millipore, Molsheim, France) or the TempliPhi DNA sequencing template amplification kit (GE Healthcare, Uppsala, Sweden). BAC Miniprep96 Kit (Millipore, Molsheim, France) was used for BAC templates. Sequencing reactions were done, from both ends of DNA inserts, using ABI PRISM BigDye Terminator cycle sequencing ready reactions kit and run on a 3700 Genetic Analyzer (Applied Biosystems, Foster City, CA, USA). The trace file was used with the Phred-Phrap-Consed package to perform the assembly [79]. Sequencing reactions were performed to close gaps, improve coverage and resolve sequence ambiguities using PCR products amplified from genomic DNA or DNA plasmid templates.

### Phylogenetic analysis

A dataset containing a concatenation of the 16S and 23S sequences was aligned by Muscle [80], and the alignment was manually edited to remove ambiguously aligned positions, giving a final dataset of 4195 nucleotide positions for phylogenetic analysis. From this dataset, a maximum likelihood tree was calculated by Phyml [81], using the HKY model of nucleotide evolution with an estimation of the transition/transversion ratio, including 4 rates of site heterogeneity, an estimated number of invariable positions, and an estimated alpha shape parameter. The numbers at the nodes correspond to the bootstrap values calculated on 1000 resampled datasets by Phyml.

### Syntenic score computation

Ten orthologs located on either side of one pair of putatively orthologous CDS (linked by BDBH) were analyzed. For each pair of orthologous genes located in the proximity of the tested gene and of its ortholog, the synteny score was incremented by 1. Using this method of calculation, two totally syntenic genomes will have a score of 20 attributed to each of their orthologs, whereas two-non syntenic genomes will have a score of 0.

### Restriction-modification enzymes

Putative restriction enzymes were identified by Blast searching of known type I and II restriction enzymes against the Mic-PCC7806 genome. Because DNA methylases are more reliably identified by Blast than restriction enzymes, we also identified all methylases, and examined the surrounding genes for potential restriction enzymes.

### Detection of atypical CDSs

A first-order Markov model was built based on the dinucleotide composition of the core genes of a group of 8 selected cyanobacterial genomes (Table 4), identified by bi-directional best hits using BLASTp (bitscore of 30% against itself). This Markov model takes into account the Markov probability matrix of the core genes to analyse whether the composition of the CDS under study is "atypical", using the formula described in [25]. For each CDS, the model calculates an index that represents the likelihood that CDS will have a dinucleotide composition compatible with that of the core genes. In order to assess significance cutoffs, we applied the following statistics [82]: for each gene analyzed, one million random sequences were generated based on the Markov model probability matrix of the core genes, and the Markov index was calculated for each of these random sequences. The results were then analyzed by a one-tailed test with cut-offs of 0.1%. The cut-off was defined after several *in silico *horizontal gene transfer simulations, during which random genes from different genomes were introduced artificially into the genome sequences under study. The optimal threshold (0.1%) was defined for all the genomes of the group as the value at which the model had the highest detection of the *in silico *introduced genes (true positives), and the lowest detection of core genes (false positives).

### Clustering of atypical genes

We defined an initial cluster of at least 4 neighboring atypical genes which was allowed to grow (in both directions) searching for other nearby atypical genes, until regions containing 4 or more non-atypical genes appeared. By this process, a reduced number of less-atypical genes and of normal genes could be included in a larger CAG.

## Abbreviations

CDS: coding sequence; HSP: high scoring segment pair; BDBH: bidirectional best hit; rDNA: ribosomal DNA; CAG: cluster of atypical gene; BV: bootstrap value. NRPS: nonribosomal peptide synthetase; PKS: polyketide synthase; N50: contig size such that all the larger contigs contain 50% of the bases of the assembly.

## Authors' contributions

LF carried out the bioinformatics studies. PQ and A–MC carried out the molecular genetic studies. AB and A–MC constructed the DNA libraries. CB, AL, PQ and A–MC carried out the sequence of the genome. LF, PQ, A–MC, NTM, ED, J–FH, J–CK, YZ, and NZ annotated the genome. HCPM, SB and NTM analyzed the metabolic pathways. DC carried out the CAG analyses. AT, PQ and LF carried out the enzyme and 6-mer analyses. SG and LF performed the phylogenetic analyses. CCZ, ET and AL analyzed the sensor and regulatory systems. NTM, LF and PQ designed the research. NTM coordinated the study. C–CZ, SG, DC, HCPM and AT drafted the manuscript. LF, NTM, J–FH, PQ and ED wrote the manuscript.

All authors read and approved the final manuscript.

## Supplementary Material

Additional file 1 Representation of the location of the genes of Mic-PCC7806-contig328 on the genome of Mic-NIES843. Click here for file

Additional file 2 Similarity of orthologous genes between Mic-PCC7806, Cwa-WH8501 and Syn-PCC6803. Click here for file

Additional file 3 Distribution of genome lengths for several cyanobacterial genomes. Click here for file

Additional file 4 Schematic representation of the phosphate transport gene cluster. Click here for file

Additional file 5 Distribution of the intergenic distances in cyanobacterial and other bacterial genomes. Click here for file

Additional file 6 Putative restriction endonucleases in the genome of Mic-PCC7806. Click here for file

Additional file 7 Putative methylases and methyltransferases in the genome of Mic-PCC7806. Click here for file

Additional file 8 Identity of the 1% 6-mers that were least common in the genome of Mic-PCC7806. Click here for file

Additional file 9 Occurrence of the putative *dnd *gene products in cyanobacteria. Click here for file

Additional file 10 Fermentation pathway adapted from Moezelaar and Stal [34]. Click here for file

Additional file 11 Genes of the circadian clock system. Click here for file

Additional file 12 Alignment of the amino acid sequences of the putative caspases from Mic-PCC7806, Mic-NIES843 and a metacaspase from *Saccharomyces cerevisiae*. Click here for file

Additional file 13 Genes of the methionine salvage pathway. Click here for file
