# Supplementary material for: Highly plastic genome of Microcystis aeruginosa PCC 7806, a ubiquitous toxic freshwater cyanobacterium
Source: BMC Genomics. 2008 Jun 5;9:274. doi: 10.1186/1471-2164-9-274 (PMC2442094; doi:10.1186/1471-2164-9-274)
Supplement: Additional file 1 — Representation of the location of the genes of Mic-PCC7806-contig328 on the genome of Mic-NIES843. [file 1471-2164-9-274-S1.pdf]

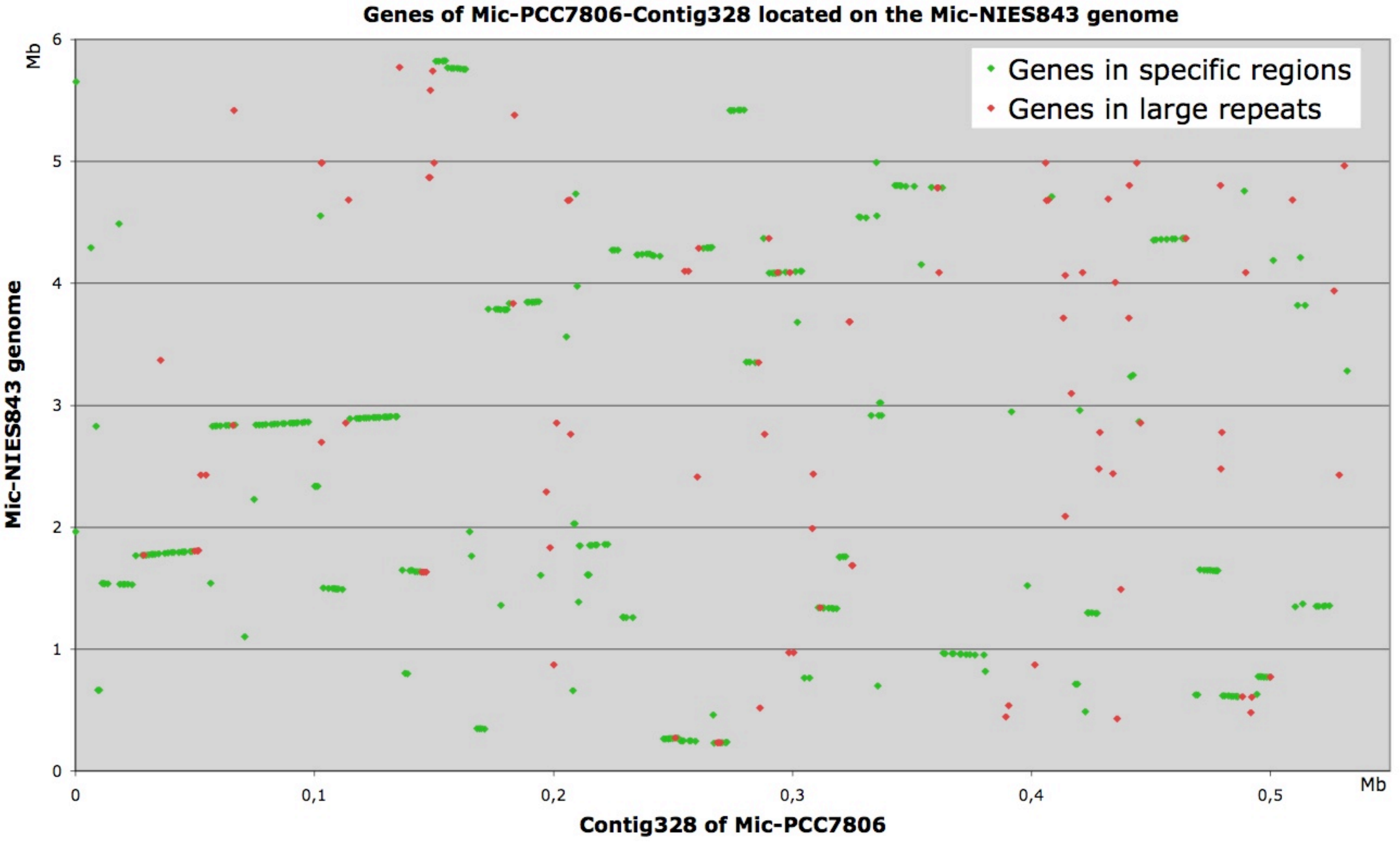

Additional file 1: Representation of the location of the genes of Mic-PCC7806 contig328 on the genome of Mic-NIES843. Two proteins are considered to be identical if they share at least 40% similarity on their total length. Large repeats are defined as DNA regions containing more than 1000 bases and a similarity threshold >90%. See the Methods section for the strain identifiers.
