# Supplementary material for: Highly plastic genome of Microcystis aeruginosa PCC 7806, a ubiquitous toxic freshwater cyanobacterium
Source: BMC Genomics. 2008 Jun 5;9:274. doi: 10.1186/1471-2164-9-274 (PMC2442094; doi:10.1186/1471-2164-9-274)
Supplement: Additional file 2 — Similarity of orthologous genes between Mic-PCC7806, Cwa-WH8501 and Syn-PCC6803. [file 1471-2164-9-274-S2.pdf]

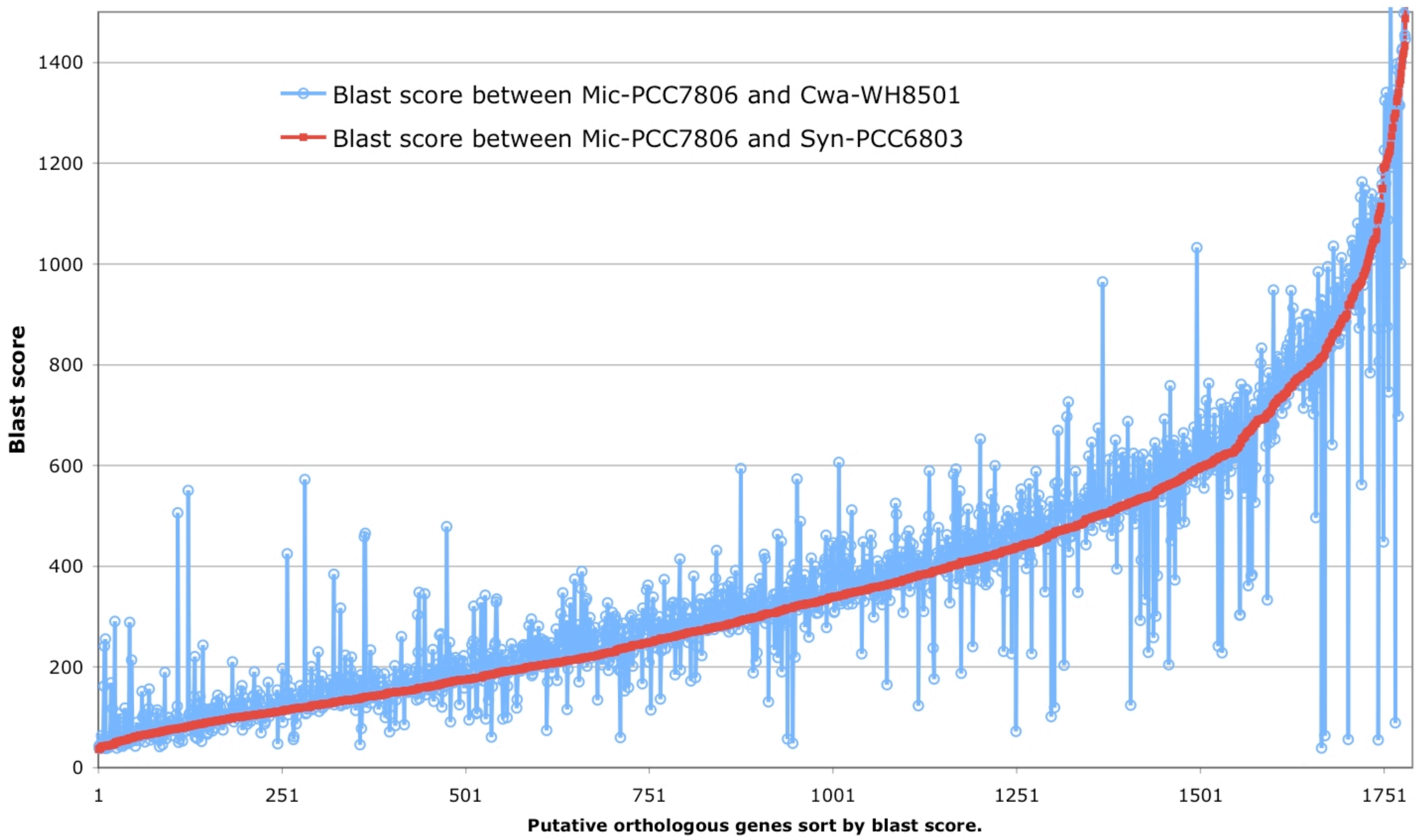

Additional file 2: Distribution of the Blastp scores among the 1789 putative orthologous CDSs of Mic-PCC7806, Cwa-WH8501 and Syn-PCC6803. Red curve: Blastp scores between Mic-PCC7806 and Syn-PCC6803; blue curve: Blastp scores between Mic-PCC7806 and Cwa-WH8501. See the Methods section for the strain identifiers.
