# Supplementary material for: Highly plastic genome of Microcystis aeruginosa PCC 7806, a ubiquitous toxic freshwater cyanobacterium
Source: BMC Genomics. 2008 Jun 5;9:274. doi: 10.1186/1471-2164-9-274 (PMC2442094; doi:10.1186/1471-2164-9-274)
Supplement: Additional file 3 — Distribution of genome lengths for several cyanobacterial genomes. [file 1471-2164-9-274-S3.pdf]

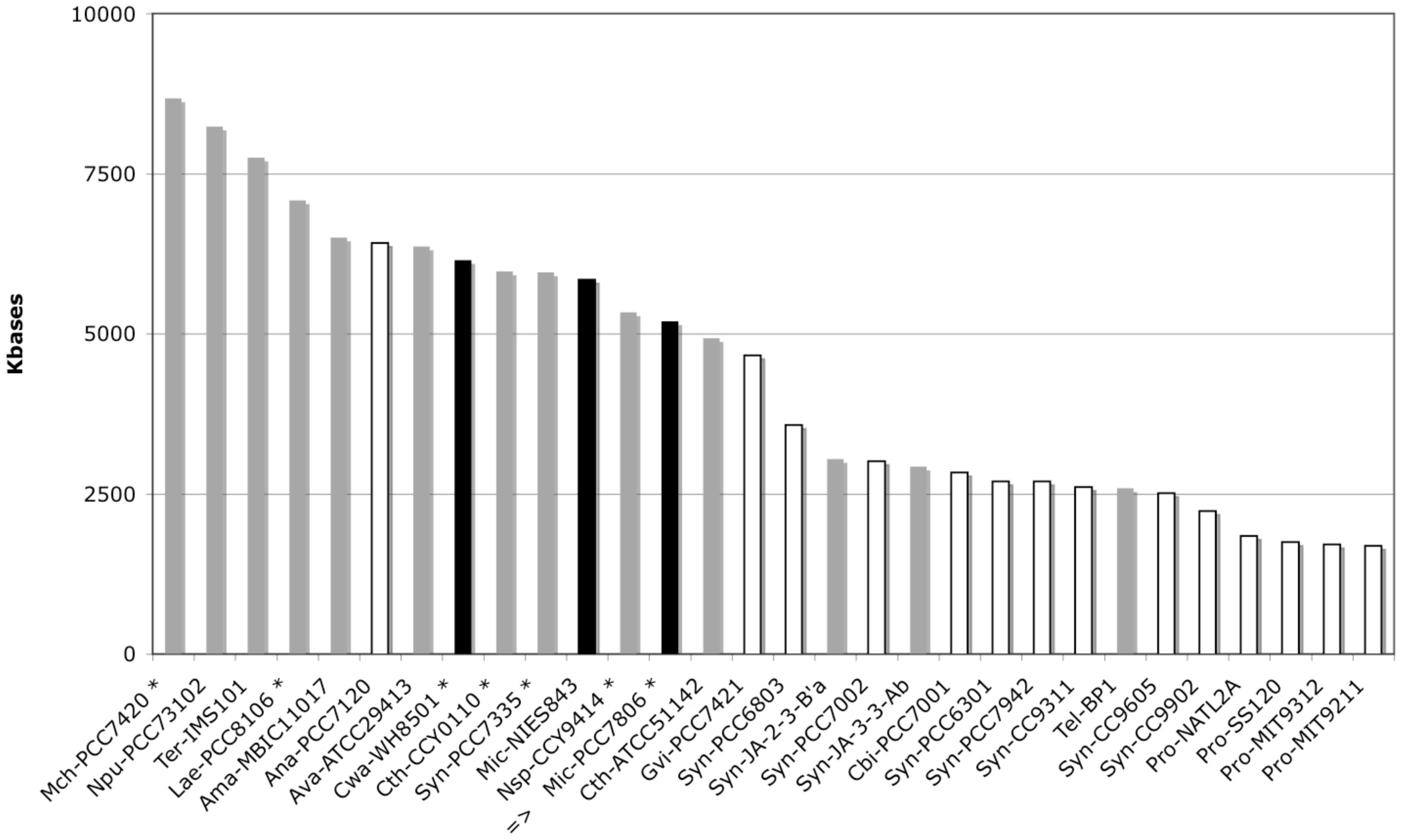

Additional file 3: Representation of the total length of cyanobacterial genomes. The filling of the histogram bars indicates the percentage of DNA repeated sequences in each genome as defined in Figure 2 (Black: >10%; grey: >1%; white: ≤1%). See the Methods section for the strain identifiers.
