# Supplementary material for: Highly plastic genome of Microcystis aeruginosa PCC 7806, a ubiquitous toxic freshwater cyanobacterium
Source: BMC Genomics. 2008 Jun 5;9:274. doi: 10.1186/1471-2164-9-274 (PMC2442094; doi:10.1186/1471-2164-9-274)
Supplement: Additional file 4 — Schematic representation of the phosphate transport gene cluster. [file 1471-2164-9-274-S4.pdf]

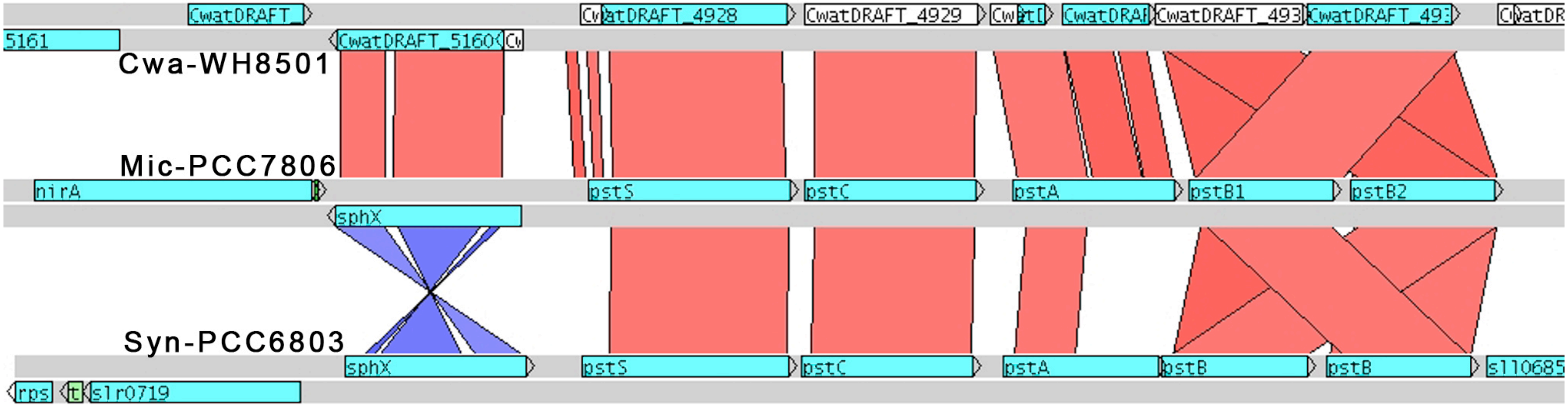

Additional file 4: Schematic representation of the phosphate transport gene cluster conserved in Mic-PCC7806, Cwa-WH8501 and Syn-PCC6803 (based on an output of ACT software; www.sanger.ac.uk/Software/ACT). See the Methods section for the strain identifiers.
