# Supplementary material for: Highly plastic genome of Microcystis aeruginosa PCC 7806, a ubiquitous toxic freshwater cyanobacterium
Source: BMC Genomics. 2008 Jun 5;9:274. doi: 10.1186/1471-2164-9-274 (PMC2442094; doi:10.1186/1471-2164-9-274)
Supplement: Additional file 5 — Distribution of the intergenic distances in cyanobacterial and other bacterial genomes. [file 1471-2164-9-274-S5.pdf]

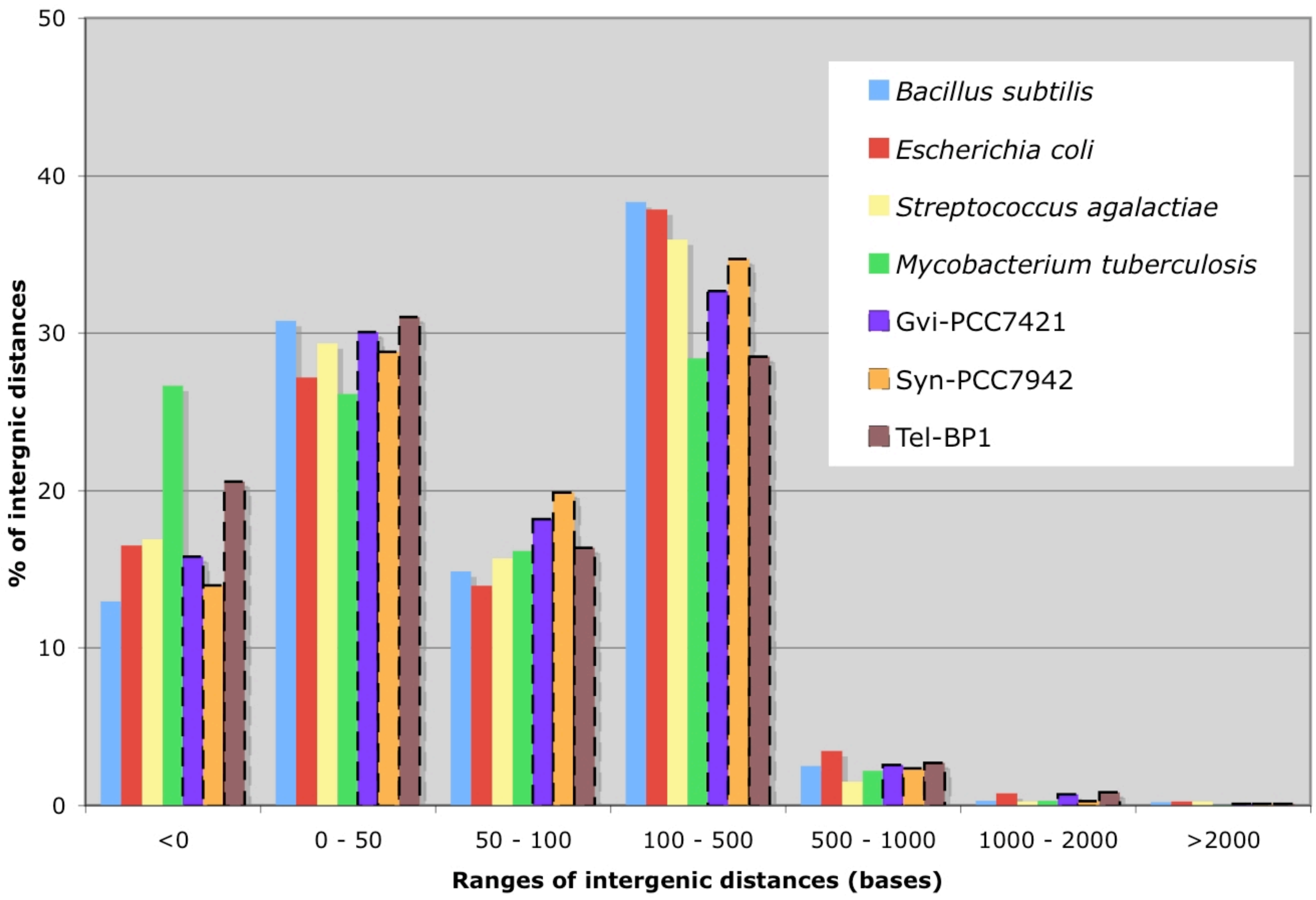

Additional file 5: Distribution of the intergenic distances in cyanobacterial and other bacterial genomes. The distances are based on the public syntaxic annotation of each genome. See the Methods section for the strain identifiers.
