# Supplementary material for: Highly plastic genome of Microcystis aeruginosa PCC 7806, a ubiquitous toxic freshwater cyanobacterium
Source: BMC Genomics. 2008 Jun 5;9:274. doi: 10.1186/1471-2164-9-274 (PMC2442094; doi:10.1186/1471-2164-9-274)
Supplement: Additional file 6 — Putative restriction endonucleases in the genome of Mic-PCC7806. [file 1471-2164-9-274-S6.pdf]

Additional file 6

Putative restriction endonucleases in the genome of *Microcystis aeruginosa* PCC 7806

Gene ID	COG number	Function
mic0099	COG3440	Predicted restriction endonuclease
mic0856	COG1715	Restriction endonuclease (Mrr restriction system protein)
mic1049	COG1403	Restriction endonuclease HNH superfamily
**mic1233**		**Type II restriction enzyme (probable endonuclease *HindVP*)**
mic1522	COG1403	Restriction endonuclease HNH superfamily
mic1606		Restriction endonuclease (possible *MspA1I*)
**mic1612**		**Restriction endonuclease (possible endonuclease *MspA1I*)**
**mic2809**		**Type II site-specific deoxyribonuclease (possible endonuclease *AvaI*)**
mic3268	COG4096	Type I/III site-specific restriction-modification system; restriction subunit and related helicase
mic3271	COG0732	Type I Restriction endonuclease; HsdS subunit
mic3701	COG4096	Type I/III site-specific restriction-modification system; restriction subunit and related helicase
**mic3705**	**COG0732**	**Type I Restriction endonuclease; HsdS subunit**
mic3707		Type I restriction enzyme M protein
mic3969	COG0732	Type I site-specific deoxyribonuclease
mic3993		Type III Restriction endonuclease; restriction subunit
mic4233		Type I restriction enzyme; HsdR restriction subunit
**mic4262**		**Type II restriction endonuclease (possible endonuclease *EcoRV*)**
**mic4389**		**Type II restriction endonuclease**
mic4421	COG1403	Restriction endonuclease HNH superfamily
**mic5299**		**Type II restriction enzyme (probable endonuclease *NspV*)**
mic6570		Type I Restriction endonuclease; HsdS subunit

Enzymes shown in bold are co-localized with putative methylases shown in the table of additional file 7.
