# Supplementary material for: Highly plastic genome of Microcystis aeruginosa PCC 7806, a ubiquitous toxic freshwater cyanobacterium
Source: BMC Genomics. 2008 Jun 5;9:274. doi: 10.1186/1471-2164-9-274 (PMC2442094; doi:10.1186/1471-2164-9-274)
Supplement: Additional file 7 — Putative methylases and methyltransferases in the genome of Mic-PCC7806. [file 1471-2164-9-274-S7.pdf]

Additional file 7

Putative methylases/methyltransferases in the genome of *Microcystis aeruginosa* PCC 7806

Gene ID	COG number	Function
mic0260	COG0270	Site-specific DNA methylase (putative *Eco47II*, *Sau96I* modification methylase)
mic0262	COG0863	DNA modification methylase
mic0319		SAM-dependent methyltransferase
mic0367		SAM-dependent methyltransferase
mic0434	COG4889	Adenine specific DNA methyltransferase
mic0510		SAM-dependent methyltransferase
mic0553		SAM-dependent methyltransferase
mic0555		SAM-dependent methyltransferase
mic0714	COG0286	Type I restriction-modification system methyltransferase subunit
mic0757		SAM-dependent methyltransferase
mic0919		Modification methylase (putative *EcoRV* methylase)
mic1132		SAM-dependent methyltransferase
mic1135		Predicted O-methyltransferase
mic1145	COG0286	Type I restriction-modification system methyltransferase subunit
**mic1231**	**COG0270**	**Site-specific DNA methylase**
mic1247		SAM-dependent methyltransferase
mic1485	COG0270	Site-specific DNA methylase
mic1536	COG0270	Site-specific DNA methylase
**mic1611**	**COG0338**	**Site-specific DNA methylase (*MspA1I* methyltransferase)**
mic1716	COG0286	Type I restriction-modification system methyltransferase subunit
mic1905		predicted N-6 adenine-specific DNA methylase
mic1963	COG0313	Predicted methyltransferase
mic2077		SAM-dependent methyltransferase
mic2080		SAM-dependent methyltransferase
mic2088	COG1002	Type II restriction enzyme, methylase subunit
mic2201	COG0270	Site-specific DNA methylase
mic2315		Site-specific DNA methylase
mic2318	COG0863	DNA modification methylase (putative *PvuII* modification methylase)
mic2460		SAM-dependent methyltransferase
mic2464	COG0863	DNA modification methylase
mic2569		SAM-dependent methyltransferase
mic2593	COG1002	Type II restriction enzyme, methylase subunit
**mic2808**	**COG0863**	**DNA modification methylase (putative *AvaI* modification methylase)**
mic3127		SAM-dependent methyltransferase
mic3146	COG0863	DNA modification methylase (cytosine methylase)
mic3149	COG0863	DNA modification methylase (putative *CfrBI* modification methylase)
mic3329	COG1092	Predicted SAM-dependent methyltransferase
mic3380	COG1743	Adenine-specific DNA methylase containing a Zn-ribbon
mic3417	COG2189	Adenine specific DNA methylase (Putative type II DNA modification enzyme)
mic3467	COG1002	Type II restriction enzyme, methylase subunit
mic3571	COG0116	Predicted N6-adenine-specific DNA methylase
**mic3707**	COG0286	**Type I restriction-modification system methyltransferase subunit**
mic3729	COG0220	Predicted SAM-dependent methyltransferase
mic3737		SAM-dependent methyltransferase
mic3776		Methyltransferase (putative *SsoI* methylase)
mic3919	COG0270	Site-specific DNA methylase (putative type II *HaeII* modification methylase)
mic4082	COG0863	DNA modification methylase (putative *BstYI* methyltransferase)
mic4091	COG1002	Type II restriction enzyme, methylase subunit
mic4194		SAM-dependent methyltransferase
**mic4261**	**COG0338**	**Site-specific DNA methylase (putative *EcoRV* modification methylase)**
mic4360		SAM-dependent methyltransferase
**mic4390**	**COG0270**	**Site-specific DNA methylase**
mic4632	COG0270	Site-specific DNA methylase
mic4878		SAM-dependent methyltransferase
mic4888	COG1002	Type II restriction enzyme, methylase subunit
mic4913	COG0286	Type I restriction-modification system methyltransferase subunit
mic4976		SAM-dependent methyltransferase
mic5066	COG0270	Site-specific DNA methylase (putative *HhaI* modification methylase)
mic5070	COG1002	Type II restriction enzyme, methylase subunit
mic5255		SAM-dependent methyltransferase
**mic5298**		**Modification methylase (putative *NspV* methylase)**
mic5314		SAM-dependent methyltransferase
mic5330		Type II restriction enzyme, methylase subunit
mic5528		N6-adenine-specific methylase
mic5612		SAM-dependent methyltransferase
mic5613	COG0338	Site-specific DNA methylase
mic5683	COG0338	Site-specific DNA methylase
mic6590	COG1002	Type II restriction enzyme, methylase subunit
mic6600	COG0270	Site-specific DNA methylase (putative *HhaI* modification methylase)
mic7256	COG0286	Type I restriction-modification system methyltransferase subunit

Enzymes shown in bold are co-localized with putative methylases shown in the table of additional file 6.
