# Supplementary material for: Highly plastic genome of Microcystis aeruginosa PCC 7806, a ubiquitous toxic freshwater cyanobacterium
Source: BMC Genomics. 2008 Jun 5;9:274. doi: 10.1186/1471-2164-9-274 (PMC2442094; doi:10.1186/1471-2164-9-274)
Supplement: Additional file 8 — Identity of the 1% 6-mers that were least common in the genome of Mic-PCC7806. [file 1471-2164-9-274-S8.pdf]

Additional file 8

Characteristics of the 1% of the rare 6-mers in the genome of *Microcystis aeruginosa* PCC 7806

6-mers	occurrence	Palindrome	Enzyme	Organism
ACATGC	15	no		
GTGCAC	17	yes	*ApaLI*	*Acetobacter*
CGTACG	18	yes	*BsiW*	*Bacillus*
ACATGT	18	yes	*PciI*	*Planococcus*
GCATGT	21	no		
GACGTC	23	yes	*AatII*	*Acetobacter*
ACGTGT	28	no		
GTCGAC	30	yes	*SalI*	*Streptomyces*
CACGTG	30	yes	*PmiI*	*Pseudomonas*
ACACGT	31	no		
GGGCAC	31	no		
TACGTA	32	yes	*SnaBI*	*Sphaerotilus*
GCTAGC	33	yes	*BmtI*	*Bacillus*
CACGTA	34	no		
GTGCTC	35	no		
GTAGAC	36	no		
GTATAC	36	yes	*BstZ17I*	*Bacillus*
GAGCTC	37	yes	*SacI*	*Streptomyces*
ACTAGT	38	yes	*SpeI*	*Sphaerotilus*
GGCGTC	38	no		
GACGCC	38	no		
GCATGC	39	yes	*SphI*	*Streptomyces*
TACGTG	44	no		
ATGCAT	45	yes	*AvaIII*	*Anabaena*
GTGCCC	45	no		
GAGCAC	47	no		
GTCTAC	50	no		
TTCGAA	55	yes	*BstBI*	*Bacillus*
GGGCTC	58	no		
GAGCCC	61	no		
TGCGCA	64	yes	FspI	*Fischerella*
GGTACC	93	yes	*Acc65I*	*Acinetobacter*
AGTACT	96	yes	*ScaI*	*Streptomyces*
CATATG	97	yes	*NdeI*	*Neisseria*
ACGCGT	98	yes	*MluI*	*Micrococcus*
CTCGAG	122	yes	*AvaI*	*Anabaena*
CGCGCG	129	yes	none	
CATACG	133	no		
CGTATG	136	no		
GGACCT	147	no		
CAGCTC	148	no		

Of the 4096 possible 6-mers, 1.5% are palindromic sequences. These sequences seem to be over-represented among the 41 rarest 6-mers in the Mic-PCC7806 genome since 51% of these 6-mers are palindromes. Most of the palindromic sequences correspond to known
