# Supplementary material for: Highly plastic genome of Microcystis aeruginosa PCC 7806, a ubiquitous toxic freshwater cyanobacterium
Source: BMC Genomics. 2008 Jun 5;9:274. doi: 10.1186/1471-2164-9-274 (PMC2442094; doi:10.1186/1471-2164-9-274)
Supplement: Additional file 9 — Occurrence of the putative dnd gene products in cyanobacteria. [file 1471-2164-9-274-S9.pdf]

Additional file 9

Occurrence of the putative *dnd* gene products in cyanobacteria

	Mch-PCC7420	Npu-PCC73102	Mic-PCC7806	Cwa-WH8501
DndE	contig24 115979 85%	NpF4494 5590866 100%	contig304 60862 87%	contig86 8211 86%
DndD	contig24 146594 74%	NpF4493 5588823 98%	contig288 3252 71%	contig200 1 43% (start contig, low quality sequence ?)
DndC	contig24 149047 81%	NpF4491 5586534 97%	contig323 2072 76%	contig209 1272 75%
DndB	contig24 151942 100% + 150729 24% sharing DGQHR motif	NpF4488 5582620 22% DGQHR motif	contig323 939 23% DGQHR motif	contig209 4042 26% DGQHR motif
DndA	contig2 93482 54% Truncated?	NpR6475 8000298 95%	contig288 15886 77%	contig118 7676 77%

	Ter-IMS101	Lae-PCC8106	Nsp-CCY9414	Ava-ATCC29413	Ana-PCC7120
DndE	ABG52813 5784151 86%	contig21 162653 76%	contig31 127021 97%	ABA21829 2744623 98%	BAB76621 5868801 97%
DndD	ABG51186 2992006 73%	contig1 1491787 73%	contig31 124232 79%	ABA21828 2742623 85%	BAB76618 5866137 85%
DndC	ABG51187 2996534 75%	contig1 1494159 74%	contig31 122544 82%	ABA21827 2740804 86%	BAB76616 5863684 84%
DndB	ABG51188 2998586 DGQHR motif	contig1 1496781 DGQHR motif	C31 120762 DGQHR motif	ABA21824 + ABA21826 2739048 DGQHR motif	BAB76615 5861928 DGQHR motif
DndA	ABG53058 6249775 78%	contig17 4899 79%	contig16 54074 84%	ABA20063 557186 86%	BAB74204 3005901 85%

	Syn-PCC6803	Sym-PCC7335	Gvi-PCC7421	Syf-PCC7942	Tel-BP1
DndE	0	0	0	0	0
DndD	0	0	0	0	0
DndC	0	0	0	0	0
DndB	0	0	0	0	0
DndA	BAA10109 2391547 65%	contig11 1341751 72%	BAC92325 4613360 68%	ABB58588 2636518 66%	BAC07668 93834 68%

Results of a filtered Blastp (E-value 1e-3) search for Dnd proteins in several genomes of cyanobacteria. Each cell contains the protein name or the contig number, the position of the CDS in the contig and the percentage of similarity with DndA-C-D-E of Npu-PCC73102 and DndB of Mch-PCC7420 (more closely related to the Dnd proteins initially described for *Streptomyces lividans*) (Zhou X, He X, Liang J, Li A, Xu T, Kieser T, Helmann JD, Deng Z:**A novel DNA modification by sulphur**, *Mol Microbiol* 2005, **57**:1428-1438).

Dark background indicates CDSs in clusters. See the Methods section for the strain identifiers.
