# Supplementary material for: Highly plastic genome of Microcystis aeruginosa PCC 7806, a ubiquitous toxic freshwater cyanobacterium
Source: BMC Genomics. 2008 Jun 5;9:274. doi: 10.1186/1471-2164-9-274 (PMC2442094; doi:10.1186/1471-2164-9-274)
Supplement: Additional file 10 — Fermentation pathway adapted from Moezelaar and Stal [34]. [file 1471-2164-9-274-S10.pdf]

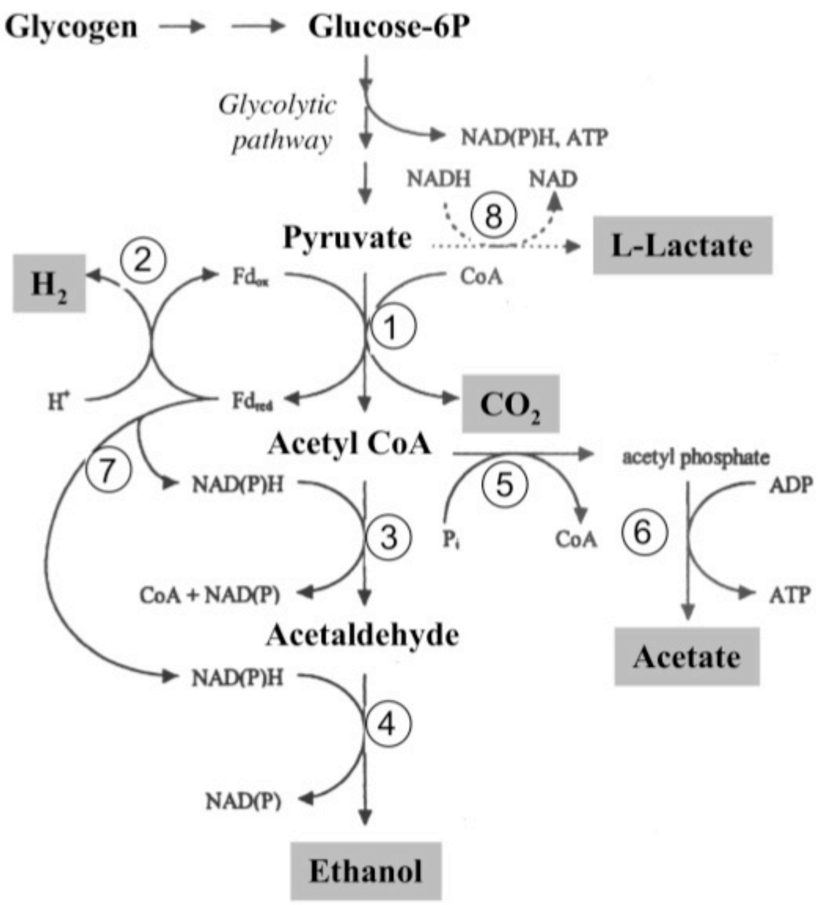

1: CoA-linked pyruvate:Fd oxidoreductase (EC: 1.2.7.1; *mic4303*)
2: Hydrogenase (EC: 1.18.99.1; *mic1925*)
3: CoA-linked aldehyde dehydrogenase (EC: 1.2.1.10; *mic0903*)
4: Alcohol dehydrogenase (EC: 1.1.1.2; *mic4903*)
5: Phosphotransacetylase (EC: 2.3.1.8; *mic3973*)
6: Acetate kinase (EC: 2.7.2.1; *mic5140*)
7: Fd:NADP oxidoreductase (EC: 1.18.1.2; *mic5476*)
8: NAD-dependent lactate dehydrogenase (EC: 1.1.1.27; *mic4831*)

Additional file 10: Fermentation pathway adapted from Moezelaar and Stal (1997). Enzyme and gene identifiers for *Microcystis aeruginosa* PCC 7806 are indicated in parentheses. Moezelaar R, Stal LJ: **A comparison of fermentation in the cyanobacterium *Microcystis* PCC 7806 grown under a light/dark cycle and continuous light**. *Eur J Phycol* 1997, **32**:373-378.
