# Supplementary material for: Highly plastic genome of Microcystis aeruginosa PCC 7806, a ubiquitous toxic freshwater cyanobacterium
Source: BMC Genomics. 2008 Jun 5;9:274. doi: 10.1186/1471-2164-9-274 (PMC2442094; doi:10.1186/1471-2164-9-274)
Supplement: Additional file 11 — Genes of the circadian clock system. [file 1471-2164-9-274-S11.pdf]

Additional file 11

Circadian clock system in *Microcystis aeruginosa* PCC 7806

Gene identifier	Gene name (a)	Putative gene product
mic0439	*kaiA*	Circadian clock protein KaiA homolog
mic0440	*kaiB1*	Circadian clock protein KaiB homolog
mic0441	*kaiC1*	Circadian clock protein KaiC homolog
mic2007	*kaiB2*	Circadian clock protein KaiB homolog
mic4842	*kaiB3*	Circadian clock protein KaiB homolog
mic2046	*kaiC2*	Circadian clock protein KaiC homolog
mic2820	*cikA*	Circadian input kinase CikA
mic3962	*sasA*	Adaptive-response sensory histidine kinase SasA
mic1620	*rpaA*	DNA binding response regulator RpaA
mic1745	*labA*	Negative regulator for circadian gene expression LabA
mic5879	*ldpA*	Circadian period regulator LdpA
mic0802	*cpmA*	Circadian phase modifier CpmA

(a) Mackey SR and Golden SS: **Winding up the cyanobacterial circadian clock.** *Trends Microbiol* 2007, **15**:381-388.
