# Supplementary material for: Highly plastic genome of Microcystis aeruginosa PCC 7806, a ubiquitous toxic freshwater cyanobacterium
Source: BMC Genomics. 2008 Jun 5;9:274. doi: 10.1186/1471-2164-9-274 (PMC2442094; doi:10.1186/1471-2164-9-274)
Supplement: Additional file 12 — Alignment of the amino acid sequences of the putative caspases from Mic-PCC7806, Mic-NIES843 and a metacaspase from Saccharomyces cerevisiae. [file 1471-2164-9-274-S12.pdf]

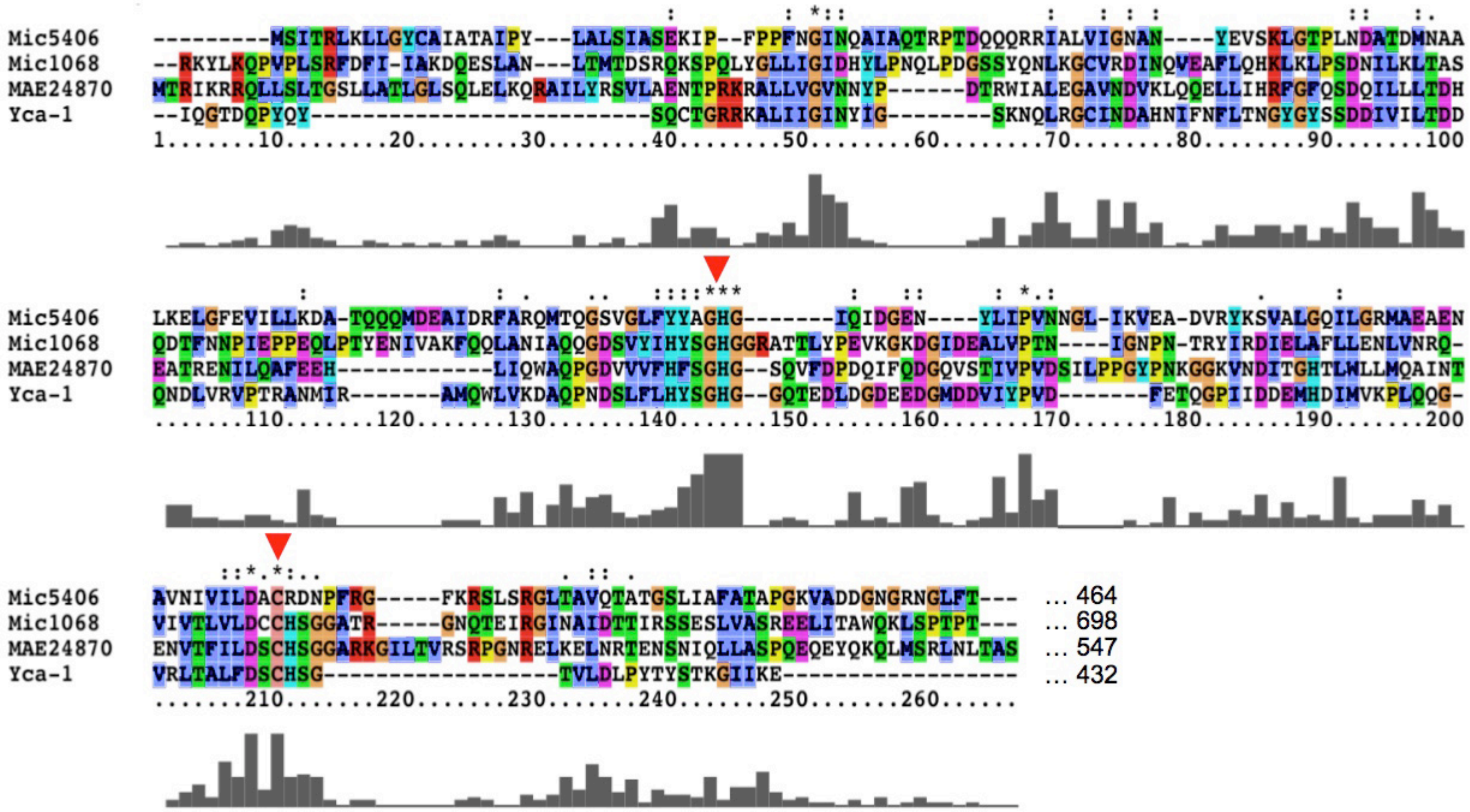

Additional file 12: Alignment of the amino acid sequences of the putative caspases from Mic-PCC7806 (Mic5406 and Mic1068), Mic-NIES843 (MAE24870) and a metacaspase from *Saccharomyces cerevisiae* (Yca-1: NP_014840). The region containing the conserved caspase domain (COG4249) was chosen for alignment. The total length of each protein is given at the end of the alignment. The alignment, done using ClutalX, shows sequence similarity and identity. The Histidine-Cysteine catalytic diad of the key functional regions of caspases are shown with red arrows. See the Methods section for the strain identifiers.
