# Supplementary material for: Highly plastic genome of Microcystis aeruginosa PCC 7806, a ubiquitous toxic freshwater cyanobacterium
Source: BMC Genomics. 2008 Jun 5;9:274. doi: 10.1186/1471-2164-9-274 (PMC2442094; doi:10.1186/1471-2164-9-274)
Supplement: Additional file 13 — Genes of the methionine salvage pathway. [file 1471-2164-9-274-S13.pdf]

Additional file 13

**Methionine salvage pathway in *Microcystis aeruginosa* PCC 7806**			
Gene identifier	Gene name (a)	EC number	Putative gene product
mic1437	*metK*	EC: 2.5.1.6	S-adenosylmethionine synthetase
mic8798	*speD*	EC: 4.1.50	S-adenosylmethionine decarboxylase
mic3610	*speE*	EC: 2.5.1.16	spermidine synthase
mic6534	*mtnP*	EC: 2.4.2.28	5'-methylthioadenosine phosphorylase
mic4478	*mtnA*	EC: 5.3.1.23	methylthioribose-1-phosphate isomerase
mic1285	*mtnB*	EC: 4.2.1.109	methylthioribulose-1-phosphate dehydratase
mic2991	*mtnW*	EC: 3.1.3.77	2,3-diketo-5-methylthiopentyl-1-phosphate enolase
mic2099	*mtnX*	EC: 3.1.3.77	2-hydroxy-3-keto-5-methylthiopentenyl-1-phosphate phosphatase
mic3733	*mtnZ*	EC: 1.13.11.54	1,2-dihydroxy-3-keto-5-methylthiopentene dioxygenase
mic4336	*mtnE*	EC: 2.6.1.-	2-keto-4-methylthiobutyrate transferase

(a) Sekowska A, Dénervaud V, Ashida H, Michoud K, Hass D, Yokota A , Danchin A:**Bacterial variations on the methionine salvage pathway**. *BMC Microbiol* 2004, **4**:1-17
